# Comparative Mitochondrial Genomics of 104 Darwin Wasps (Hymenoptera: Ichneumonidae) and Its Implication for Phylogeny

**DOI:** 10.3390/insects13020124

**Published:** 2022-01-25

**Authors:** Boying Zheng, Yuanyuan Han, Ruizhong Yuan, Jingxian Liu, Cornelis van Achterberg, Pu Tang, Xuexin Chen

**Affiliations:** 1 State Key Laboratory of Rice Biology, Zhejiang University, Hangzhou 310058, China; zhengboyingg@163.com (B.Z.); yyhan6@zju.edu.cn (Y.H.); monareason@zju.edu.cn (R.Y.); kees@vanachterberg.org (C.v.A.); xxchen@zju.edu.cn (X.C.); 2 Institute of Insect Sciences, College of Agriculture and Biotechnology, Zhejiang University, Hangzhou 310058, China; 3 Department of Entomology, College of Plant Protection, South China Agricultural University, Guangzhou 510642, China; jingxianliu@foxmail.com; 4 Ministry of Agriculture Key Laboratory of Molecular Biology of Crop Pathogens and Insects, Zhejiang University, Hangzhou 310058, China; 5 Zhejiang Provincial Key Laboratory of Biology of Crop Pathogens and Insects, Zhejiang University, Hangzhou 310058, China

**Keywords:** mitochondrial genome, gene rearrangement, ichneumonid wasps, phylogeny, divergence

## Abstract

**Simple Summary:**

Nearly a hundred mitochondrial genomes of ichneumonid wasps are newly reported. Comparative mitogenomics of 104 mitochondrial genomes representing 33 subfamilies of Ichneumonidae, as well as its implications for phylogeny, were studied. We found that the mitochondrial genomes of ichneumonid wasps were highly conserved in their base composition and had low evolutionary rates, but were diverse in gene order. There are 38 types of gene rearrangement events in 104 ichneumonid mitochondrial genomes, of which 30 novel rearrangement types (R3-6, R8-R10, R12-R15, R17-R18, R20-R35 and R38) and a hot spot rearrangement around R1, with a shuffled tRNA cluster *trnW-trnY-trnC* and *trnI-trnQ-trnM*, were detected. The relationships among these subfamilies are firstly discussed based on mitochondrial genomes at a large scale. We suggest five subfamily groupings of Ichneumonidae: Brachycyrtiformes, Ichneumoniformes, Ophioniformes, Pimpliformes and Xoridiformes. Two formerly unplaced subfamilies, Eucerotinae and Microleptinae, were placed in Brachycyrtiformes and Ichneumoniformes, respectively.

**Abstract:**

Ichneumonidae is one of the largest families of insects with a mega-diversity of specialized morphological and biological characteristics. We newly sequenced 92 mitochondrial genomes of ichneumonid wasps and found that they have a conserved base composition and a lower evolutionary rate than that of other families of parasitic Hymenoptera. There are 38 types of gene order in the ichneumonid mitochondrial genome, with 30 novel types identified in 104 ichneumonids. We also found that the rearrangement events occur more frequently in Ophioniformes than in Ichneumoniformes and Pimpliformes. Furthermore, the higher Ophioniformes and their relative lineages shared the transposition of *trnL2* to *trnI-trnQ-trnM* tRNA cluster. We confirmed five higher-level groupings of Ichneumonidae: Brachycyrtiformes, Ichneumoniformes, Ophioniformes, Pimpliformes and Xoridiformes. Two formerly unplaced subfamilies, Eucerotinae and Microleptinae, were placed in Brachycyrtiformes and Ichneumoniformes, respectively. The results will improve our understanding of the diversity and evolution of Ichneumonidae.

## 1. Introduction

The family Ichneumonidae (Insecta: Hymenoptera) is one of the most species-rich families of insects, with approximately 25,000 described species, and many more are likely to be found [1,2,3]. Ichneumonidae, or Darwin wasps, have a mega-diversity of specialized morphological and biological characteristics, and a world-wide distribution [4]. They parasitize holometabolous insects and occasionally spiders, and their types of life history are diversified, including ecto- and endoparasitoism and idiobiont/koinobiont strategies [5,6]. Ichneumonids are economically important because they can be used as biological control agents against agricultural pests [4,6]. Many researchers have strived to explore the well-inferred phylogenetic tree to understand the ecology, evolution and diversification patterns of the Ichneumonidae family.

The earliest high-level groupings of Ichneumonidae were proposed based on larval morphology. Ichneumoniformes [7,8], Ophioniformes [9] and Pimpliformes [10] were recognized as the main high-level groupings of Ichneumonidae. Subsequently, most studies focus on the sequence of a single gene (usually 28S rRNA) or combined morphological and biological characteristics to explore subfamily relationships. Quicke et al. [2,11,12] combined the morphological characteristics and the sequences of the 28S rDNA region first, and explored the phylogenetic relationships among 38 subfamilies. The three morphological high-level groupings were confirmed and four other groupings (Xoridiformes, Labeniformes, Orthopelmatiformes and Brachycyrtiformes) were defined. Bennett et al. [13] examined the phylogeny of the whole family extensively by using the morphological characteristics and three genes, and constructed the most detailed analyses to date. Recently, Klopfstein et al. [14] and Sharanowski et al. [15] used 93 and 541 nuclear genes, respectively, to discuss many inconclusive high-level relationships of the ichneumonid phylogeny. The phylogeny of Ichneumoniformes was clarified by combining nuclear genes and morphological characters [16] and genomic ultra-conserved elements (UCEs) [17] with the newly raised subfamilies, Phygadeuontinae and Ateleutinae. At present, Ichneumonidae have been grouped into 42 subfamilies [12,15,16], and three main higher-level groupings, Ichneumoniformes, Ophioniformes and Pimpliformes, were defined. The relationship of (Ophioniformes + (Ichneumoniformes + Pimpliformes)) has been proved in many studies [13,14,15,18]. However, the relationships among some members of these groups remain controversial. Xoridinae is proposed as the sister lineage to the rest of Ichneumonidae in most studies [2,14,15,19]. Regrettably, some rare lineages, such as Brachycyrtinae, Eucerotinae, Labeninae and Orthopelmatinae, were frequently absent in the above research. Obviously, the relationships among all subfamilies and higher-level groupings of Ichneumonidae need more supporting evidence.

Mitochondrial genomes were instrumental in the early definition of genome-level characteristics in phylogenetic analysis, a method that is still applied widely in insect studies [20,21,22,23,24]. Hymenoptera have been extensively sequenced and have informative rearrangement events in mitochondrial genomes [25,26,27,28,29,30]. In addition, the rearrangements of mitochondrial genes are useful clade markers in Hymenoptera [26,28,29]. The main limitations for mitochondrial genomes are that only thirteen single-copy protein genes are available, and they have a relatively high evolutionary rate, as well as the presence of base composition bias [31,32,33]. In those phylogenetic studies of deep lineages (e.g., Chrysomelidae) in insects, the dense sampling and sequencing of mitochondrial data remains a useful strategy [22,23,24]. On the other hand, Ichneumonidae have a low evolutionary rate in the generally accelerated protein-coding genes of Hymenoptera [29,32,33], and some studies on the comparative mitochondrial genomics of Braconidae (Ichneumonoidea) showed a reversed strand asymmetry of base composition [34,35]. However, until now, the mitochondrial genomes of Ichneumonidae have rarely been sequenced, and only 11 complete or nearly complete mitochondrial genomes of ichneumonid wasps are available in GenBank (up to 30 September 2020), and they represent only five of the forty-two widely well-defined Ichneumonidae subfamilies [6], which hindered the exploration of the mitochondrial features and phylogeny of this family.

In the present study, we sequenced 92 mitochondrial genomes from Ichneumonidae. The features of the mitochondrial genomes in Ichneumonidae were analyzed, including base composition and codon usage bias, the evolutionary rate of protein-coding genes and a gene rearrangement of the whole mitochondrial genome. The phylogenetic relationships within Ichneumonidae were reconstructed via protein-coding gene sequences. Our analyses provide full mitochondrial features and help to confirm previous phylogenetic uncertainties in Ichneumonidae.

## 2. Materials and Methods

### 2.1. Sampling and Species Identification

We sampled 96 ichneumonid species for mitochondrial genome sequencing. The species were identified by Jing-xian Liu and Yuan-yuan Han on the basis of the adult morphology. The subfamily classification system mainly follows that of Broad et al. (2018) [6]. All specimens were preserved in 100% ethanol and stored at 4 °C before DNA extraction. Detailed information for all the samples is listed in Table S1, and this includes the voucher specimen numbers, collection localities, GenBank accession numbers and published references.

### 2.2. DNA Extraction and Sequencing

Whole genomic DNA was extracted from each individual sample separately without destroying their surface morphology using a DNeasy^®^ Blood and Tissue Kit (Qiagen, Hilden, Germany) [36,37]. Voucher specimens are deposited in the Institute of Insect Sciences, Zhejiang University (for voucher specimen numbers, see Table S1). Extracted genomic DNA were qualified with a Qubit 3.0 (Invitrogen, Life Technologies, Carlsbad, CA, USA). The DNA libraries with single individuals (500 ng genomic DNA) were constructed using the VAHTS™ Universal DNA Library Prep Kit following the manufacturer’s protocols. The indexed libraries were sequenced using an Illumina NovaSeq sequencer with a Novogene (Beijing, China) and approximately 2 Gb of raw reads (paired-end reads) were obtained.

### 2.3. Mitochondrial Genome Assembly and Gene Annotation

Extraction and assembly of the mitochondrial reads were conducted as follows. FastQC v0.11.9 was used to check the quality of the data, and Trimmomatic v0.39 was used with default parameters to trim adaptors and indices [26]. The target mitochondrial reads were filtered out using BLAST v2.9.0+ (BLASTn, E-value cutoff 1 × 10^−5^) against a reference dataset of published Ichneumonidae mitochondrial genomes [38]. The mitochondrial reads were assembled using Spades v3.0 with default parameters [39].

The assembled contigs were annotated using the MITOS WebServer (http://mitos.bioinf.uni-leipzig.de/index.py, Access date: 12 November 2019). The start and stop codons of protein-coding genes (PCGs) were adjusted manually in Geneious Prime v11 by referencing the published Ichneumonidae mitochondrial genomes. The locations of tRNA genes were confirmed using the tRNAscan-SE online server [40]. The newly sequenced mitochondrial genomes have been submitted to GenBank (for accession numbers, see Table S1).

### 2.4. Comparative Analyses of the Mitochondrial Genomes

We analyzed the characteristics of the mitochondrial genomes, including base composition, codon and amino acid usage, and compared them with Braconidae and other parasitoid families (Table S2). The base composition was calculated with a MEGAX [41]. Nucleotide skews were calculated using the method of Perna and Kocher (1995) [42]. AT skew and GC skew were calculated as: AT skew = (A − T)/(A + T) and GC skew = (G − C)/(G + C). For neutrality plot mapping analysis, GC12 (the mean of G + C content for the 1st and 2nd positions of 13 PCGs) works as the ordinate, while GC3s (the mean of G + C content for the 3rd position of 13 PCGs) works as the abscissa. The sequences with the removed terminal codon were aligned via MUSCLE translation alignment (Genetic code: invertebrate mitochondrial) in Geneious Prime v11. The codon and amino acid usage bias, as well as the correspondence analysis (COA), were calculated with a CodonW v1.4.4 (written by Peden J.F., University of Nottingham, UK; http://codonw.sourceforge.net, Access date: 24 June 2020). The expected value of ENc under random codon usage can be expressed as: ENc = 2 + GC3s + (29/ (GC3s + (1 − GC3s)^2^)). In the present work, the codon correspondence analysis has been performed on relative synonymous codon usage (RSCU) values to minimize the effects of amino acid composition. To explore the possibilities of shaping the codon and amino acids and their usage variation among the species in Ichneumonidae and Braconidae, we have subjected the data to multivariate statistical analysis. The RSCU value variation was plotted in a multidimensional space of 61 axes (all codons in the mitochondrial genomes of insects) and the amino acids in a space of 20 axes (all amino acids in the mitochondrial genomes of insects). The same correspondence analysis was used to explain the variation of codon and amino acids and their usage within Ichneumonidae for assessing the phylogenetic fitness. The visualization of these results mentioned above was performed with an R package, ggplot2 [43].

For testing the substitutional saturation of Ichneumonidae mitochondrial protein-coding genes, the pairwise distance (transitions, ts; transversions, tv; ts and tv) was computed with a p-distance model in MEGAX [41]. The results were plotted as ts and tv for each pair of taxa against the total p-distance (ts and tv) and fitted with a 2nd order polynomial regression line. The non-synonymous substitution rate (dN) and the synonymous substitution rate (dS) for 4225 codons from all PCGs were calculated on the website Datamonkey (http://www.datamonkey.org/, Access date: 14 February 2020) based on the Single-Likelihood Ancestor Counting (SLAC) model with a *p*-value threshold of 0.1 [44].

The gene rearrangements of 104 Ichneumonidae species were compared with each other and categorized into 38 types of rearrangement by comparing them with the ancestral mitochondrial genome of the insects. Finally, the phylogenetic signal in the structure of mitochondrial genome was assessed by screening for gene rearrangements.

### 2.5. Phylogenetic Analyses

The mitochondrial genomes of the 104 ichneumonid species and eight outgroup species from the families Braconidae, Gasteruptiidae, Aulacidae and Trigonalyidae were used to reconstruct the phylogenetic relationships within Ichneumonidae. The 13 protein-coding genes were realigned using the G-INS-i algorithm implemented in MAFFT v7.464 [45,46]. We partitioned the amino acid (AA) and nucleotide (NU) matrices according to the 13 protein-coding genes and the coding sites of these genes, respectively (Table S3). The ambiguously aligned positions were identified with Aliscore v2.2 [47,48] and removed with Alicut v3.2 [49] to reduce noise. Finally, the heterogeneity of the AA matrix and NU matrix were tested using Aligroove v1.0.5. The best-fit models for the partitions of the AA and NU matrices were selected using the Bayesian Information Criterion in ModelFinder [50] (see results in Table S3) and the best-fit model for the entirety of both matrices was also assessed for Bayesian inference.

The Bayesian inference (BI) was calculated in Exabayes v1.5.1 [51]. According to best-fit models selected from Model Finder [50], the GTR + I + G and MTART + I + G models were used for the NU and AA matrices, respectively, following four independent runs with four coupled MCMC chains each. After 10 million generations of the NU matrix and 8 million generations of the AA matrix, the convergence of the results was assessed according to their effective sample size (ESS) in Tracer v1.7. [52]. The ESS values were over 200 in all runs, which is generally considered an acceptable convergence according to the ExaBayes manual. The consense tool (part of the ExaBayes software package) was used to obtain an MRE consensus tree, after discarding the first 25% of the sampled topologies. The maximum likelihood (ML) inference were conducted in IQtree v2 [53]. ML trees were constructed with best-fit models for the partitions from ModelFinder with 5 million replicates of ultra-fast bootstrapping for node support [54].

The conflicting nodes in our trees generated with different methods and matrices were examined using the likelihood mapping analyses in IQtree v2 [53,55]. For the mapping analysis, the relative 4 taxon clusters containing for the conflicting nodes were selected, and the sole likelihood mapping analyses (ignoring the tree search) were performed with 3000 quartets (about 30 times the number of 112 sequences in both two matrices) with models from ModelFinder, outlined above (Table S3).

## 3. Results

### 3.1. General Features of Ichneumonid Mitochondrial Genome

We newly sequenced 92 mitochondrial genomes from the taxa of Ichneumonidae. The 13 typical protein-coding genes (PCGs) of animal mitochondrial genome were identified in the 87 genomes, except for five that failed to sequence *nad2* (Figure S1). Only one sequence (*Sussaba sugiharai*) with nine PCGs was partial. There are 70 sequences with two rRNA genes and 22 sequences lacking rRNA genes, *rrnL* or *rrnS*, or both. Most tRNA genes are contained in our sequences, and the details are described in the gene rearrangement section.

**Figure 1:**
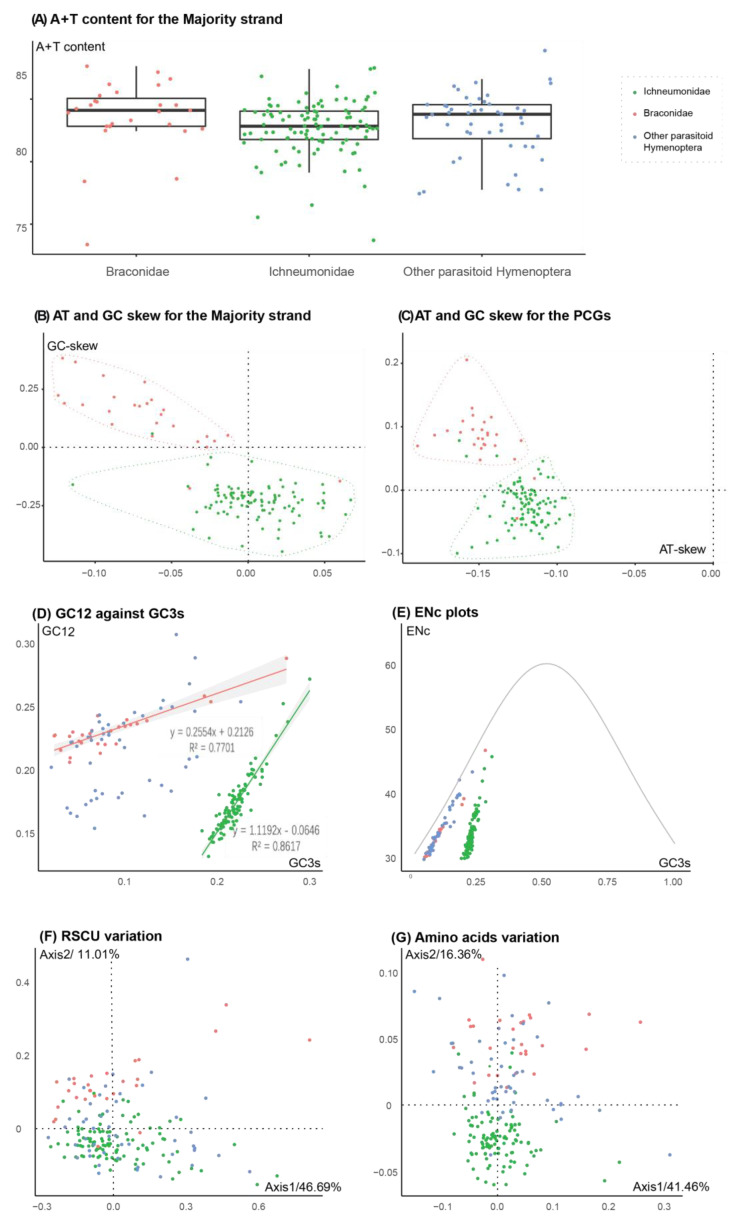
Base composition of the majority strand and the protein-coding genes (PCGs) of mitochondrial genomes (**A**–**D**) and the codon and amino acid usage bias in PCGs (**E**–**G**) for Ichneumonidae, Braconidae and other parasitoid Hymenoptera. (**A**) A + T content of the majority strand. (**B**) GC skew against AT skew of the majority strand. (**C**) GC skew against AT skew of PCGs. (**D**) GC12 against GC3s values of PCGs and the regression lines and equations with R values. (**E**) The ENc value against GC3s values and the standard curve representing the functional relation between ENc and GC3s under mutation pressure without selection. (**C**) The RUSC variation on the two principal correspondence analysis axes. The total inertia is 0.073018. The explanation of the variation of Axis1 is 46.69% and Axis2 is 11.01%, and others are lower than 5%. The abnormal species are demonstrated by their position on Axis1. (**D**) The amino acid usage variation on the two principal correspondence analysis axes. The total inertia is 0.008047. The explanation of the variation of Axis1 is 41.46% and Axis2 is 16.36%, and others are lower than 13%.

For A + T content, there were no significant differences among most species of Ichneumonidae. Most species in Ichneumonidae have a high A + T content (>80%) (Figure 1A). The GC skew of the mitochondrial majority strand was negative in Ichneumonidae (excluding *Klutiana* sp.) (Figure 1B). In addition, the base composition of the PCGs was similar to the majority strand (Figure 1C and Figure S2A,C). The G + C content for the first + second sites and third sites in the PCGs were correlative in Ichneumonidae (Figure 1D), and the trendline slope was 1.12 (near to 1), which indicated that there is little difference in the mutations between the first + second sites and third sites in ichneumonid mitochondrial genes.

In the correspondence analyses, the first two principal axes were determined, contributing to the relative synonymous codon (RSCU; Axis1: 46.69% and Axis2: 11.01%; Figure 1F) and amino acid (Axis1: 41.46% and Axis2: 16.36%; Figure 1G) usage variation in parasitoid Hymenoptera. It was obvious that the majority of the points of Ichneumonidae were clustered in a spherical shape around the origin of the axes. It was indicated that species across parasitic Hymenoptera had similar codon and amino acid usage biases. In the ENc-plots, the standard curve showed the functional relationship between ENc and GC3s was under mutation pressure rather than selection. The plots near to the curve indicated that the mutation has the main power to shape the codon bias.

**Figure 2:**
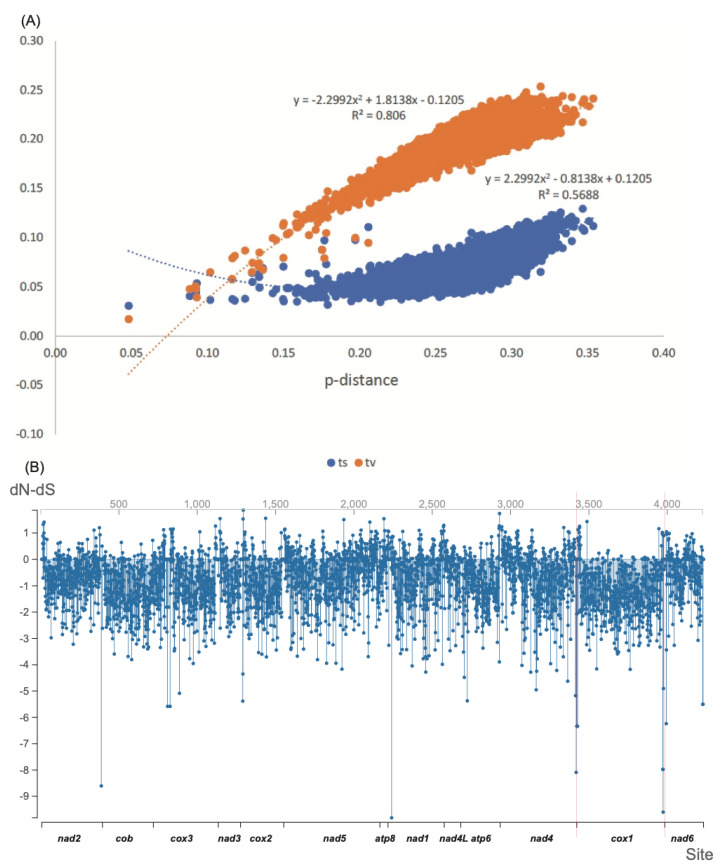
The evolution per site for 13 protein-coding genes (PCGs). (**A**) The base substitutional saturation plots. The transitions (ts, blue) and transversions (tv, orange) against p- distance for both of them. The sites included were three codon positions of PCGs. (**B**) The average dN-dS is the plot per site of 4225 PCGs.

The pairwise distance for PCGs was shown using saturation plots (Figure 2A and Figure S3). The rate of transitions was much faster than transversions, as is normal. The substitution of PCGs was slightly saturated, and this was mainly caused by the third site, as shown by comparing the slope of the line for three sites. The evolutionary rates were represented using average dN-dS with an extremely low dN/dS ratio of 0.130. We found that 2505 sites were significant at *p*-value ≤ 0.1, with a diversifying selection at 73 sites of dN-dS > 0, and a purifying selection at 2432 sites of dN-dS < 0 (Figure 2B).

### 3.2. Gene Rearrangement

**Figure 3:**
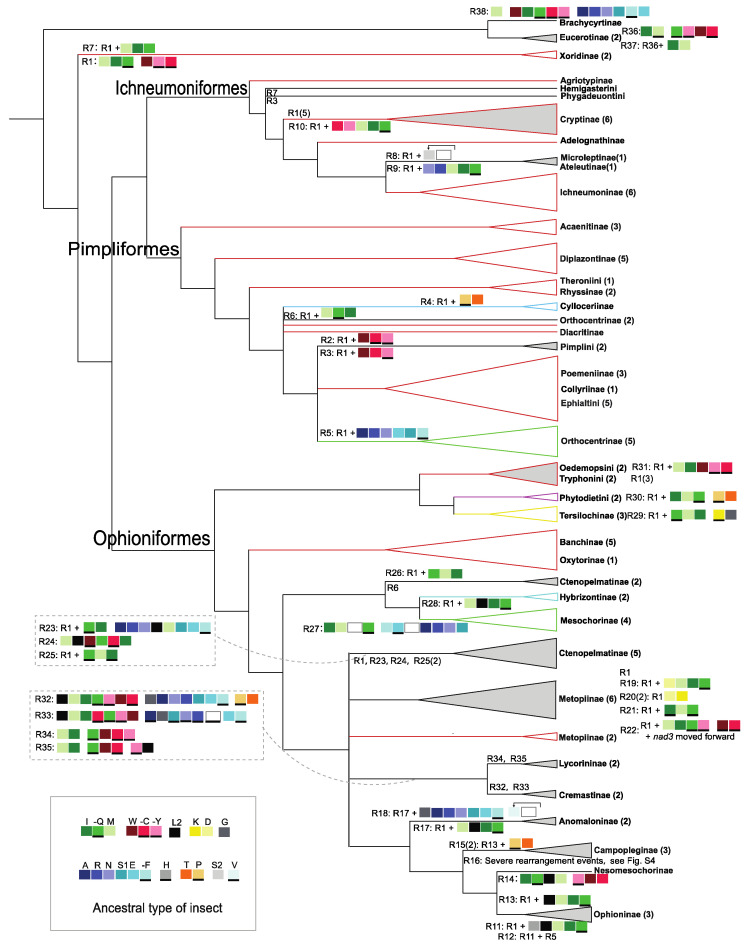
All types of gene rearrangement in the Ichneumonidae mapping of the consensus tree mainly based on AA matrices. The tRNA genes were colored out and the lines in the bottom of gene blocks indicate the gene coding on the minority strand. The ancestral type of tRNA gene clusters for insect is in the box at the lower right corner. The triangles represent the stable monophyletic groups, of which the triangles filled in gray indicate that the group has more than one type of gene rearrangement. The numbers of species are in the brackets. The genes rearrangement types are near to the corresponding branch, and the branches with R1 (rearrangement type 1) are colored in red. The complete gene orders for each species are in Figure S5 (types: R1–R38).

Compared with the putative ancestral mitochondrial genome of the insects, the mitochondrial genomes of all the ichneumonids in this study were tRNA rearranged, and no rearrangement of the protein-coding genes was detected, except for *Metopius* sp. and *Venturia canescens*. Overall, the rearrangement events were diverse, with 38 types (R1- R38) in 104 species, of which there were 30 novel rearrangement types (Figure S1). The most widespread type of rearrangement, R1, had two tRNA gene shuffles: the *trnW*-*trnC*-*trnY* block was shuffled as *trnW*-*trnY*-*trnC* and the *trnI*-*trnQ*-*trnM* block was shuffled as *trnM*-*trnI*-*trnQ*, which exist extensively in most lineages except for Brachycyrtiformes (Figure 3). The type of rearrangement, R1, occurred 35 times in Ichneumoniformes and Pimpliformes, and 11 times in Ophioniformes. Ophioniformes had more diverse rearrangement events and two species, *Venturia canescens* and *Metopius* sp., had the transposition of PCGs. All species in higher Ophioniformes shared a specific transposition of gene *trnL2* to the cluster *trnM-trnI-trnQ* (R11–R18). Interestingly, Hybrizontinae (R28) and Cremastinae (R32, R33), the subfamilies with variable placements in Ophioniformes, also had this transposition.

### 3.3. Utility of Data Matrices for Phylogenetics

We built two matrices, which have a total of 112 taxa and 13 PCGs, with lengths of 12,195 base pairs and 3425 amino acids for the AA and NU matrices, respectively. No taxa were highlighted by the test in both matrices by Aligroove (Figure S4), and no taxa have unusual codon and amino acid usage except for Hybrizontinae (Figure S5), which indicated that the lack of a confounding signal can mislead the tree reconstruction at the major lineages in the AA and NU matrices. These can help to avoid the invalid signals of the independently evolved mitochondrial genomes and make the results more reasonable.

**Figure 4:**
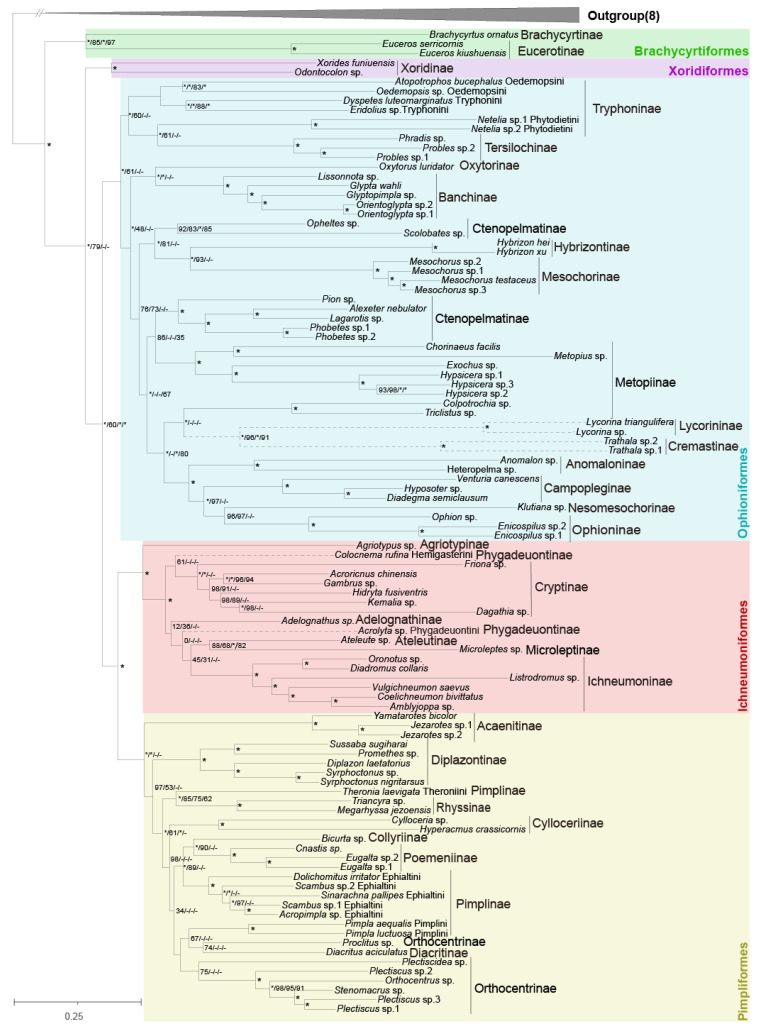
Phylogenetic relationships of Ichneumonid wasps. The topology is inferred from amino acid sequences of 13 protein-coding genes in mitochondrial genomes (AA matrices) using BI. The numbers close to the nodes separated by “/” represent the ultrafast bootstrap values and Bayesian posterior probabilities in different analysis, respectively. Additionally, “*” represents the full support and “-” represents nonsupport of the corresponding node by that analysis. The order of value for the corresponding analysis is AA by BI/AA by ML/NU by BI/NU by BI. The large “*” alone stands with full supports of all analyses. The triangles on the left confirm some conflicts nodes on the tree through likelihood mapping analysis. The relationship on the top of each triangle is supported by the colored area.

**Figure 5:**
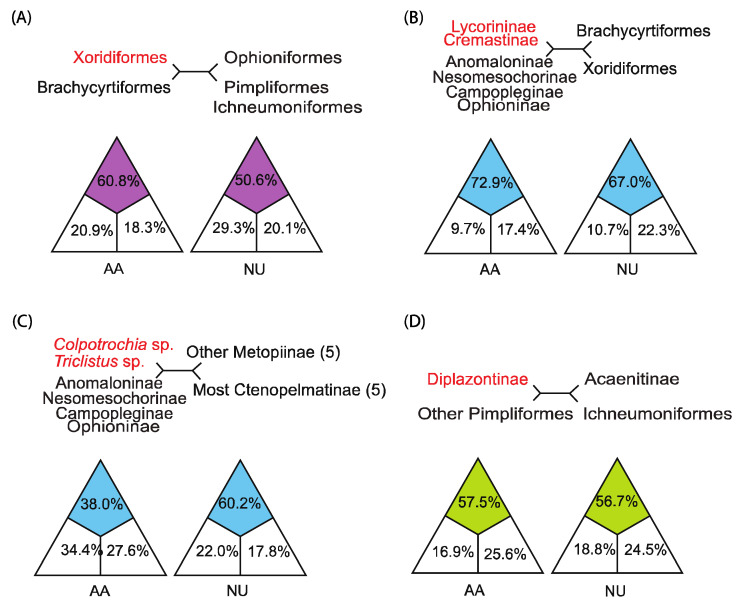
Results from likelihood mapping based on both AA and NU matrices. For analysis of conflict nodes, the conflict nodes in tests are at the top corners of rectangles. The relationship on the top of each triangle is supported by the colored area. (**A**) Test for the placement of Xoridi-formes, which indicates that Xoridiformes is close to Brachycytiformes by both AA and NU ma-trices. (**B**) Test for the placement of group (Lycorininae + Cremastinae), which indicates that group (Lycorininae + Cremastinae) is close to group (Anomaloninae + Nesomesochorinae + Campopleginae + Ophioninae) by both AA and NU matrices. (**C**) Test for the placement of group (*Colpotrochia* sp. + *Triclistus* sp.), which indicates that group (*Colpotrochia* sp. + *Triclistus* sp.) is close to group (Anomaloninae + Nesomesochorinae + Campopleginae + Ophioninae) by both AA and NU matrices. (**D**) Test for the placement of Diplazontinae, which indicates that Diplazonti-nae is close to the Pimpliformes excepting for Acaenitinae by both AA and NU matrices.

The phylogenetic relationships within Ichneumonidae were inferred based on the NU and AA matrices. The trees based on the AA matrix showed the stronger support values at most nodes, despite some inconsistent topology (Figure 4 and Figures S6). Additionally, the results from the likelihood mapping analyses verified that the topologies from the AA matrix were more reasonable (Figure 5). The trees based on the NU matrix using different methods have more incongruence and lower support values than the trees based on the AA matrix (Figures S7 and S8). All results of these analyses for the phylogeny within Ichneumonidae are presented as follows.

### 3.4. The Mitogenomic Implication of Ichneumonid Phylogeny

Ichneumonidae was strongly recovered with five higher-level groupings in all analyses: Brachycyrtiformes, Ichneumoniformes, Ophioniformes, Pimpliformes and Xoridiformes (Figure 4). Brachycyrtiformes, including subfamilies Eucerotinae and Brachycyrtinae, was sister to all remaining ichneumonids with maximal supports. Pimpliformes and Ichneumoniformes formed a monophyletic group that was sister to Ophioniformes in all analyses. In our analyses, Xoridiformes was sister to ((Pimpliformes + Ichneumoniformes) + Ophioniformes), supported strongly by the AA matrix (Figure 4 and Figure S6), but close to (Pimpliformes + Ichneumoniformes), according to the NU matrix (Figures S7 and S8). This conflict was examined with the likelihood mapping analyses, which supported the results from the AA matrix with support values of 60.8% (AA matrix) and 50.6% (NU matrix) (Figure 5A).

Pimpliformes included nine subfamilies, but the topology within Pimpliformes was equivocal (Figure 4). Diplazontinae and Acaenitinae were grouped into a monophyletic group based on the NU matrix (Figures S7 and S8). However, the topology, Acaenitinae + (Diplazontinae + other Pimpliformes), was proposed by the AA matrix, which was also confirmed by the likelihood mapping analyses (Figure 5D). Pimplinae, Theroniini, the newly resurrected tribe in Pimplinae [14], was separated out and became sister group to Rhyssinae with strong support. Ephialtini was loosely close to the ectoparasitoid Poemeniinae rather than the endoparasitoid Pimplini. However, the mapping analyses supported the monophyletic group of Pimplini and Ephialtini. *Proclitus* sp. and *Pectiscidea* sp. were separated out from Orthocentrinae, and were distantly related to most Orthocentrinae in our analyses, but their placements were variable (Figure S9). The placement of Collyriinae, Pimplinae, Cylloceriilnae and Orthocentrinae within Pimpliformes remained uncertain, because the placements heavily depend on the matrices.

Ichneumoniformes was recovered with maximal support and consisted of seven subfamilies (Figure 4). Our analyses strongly suggested that Agriotypinae was sister lineage to the rest of the Ichneumoniformes. Microleptinae, a previously unplaced subfamily, was positioned far away from the other dipteran parasites (e.g., Diplazontinae) and close to the subfamily Ateleutinae. *Colocnema rufina* (Hemigasterini) were separated from Phygadeuontinae and became the sister group to Ichneumoninae in some analyses (Figure 4), but the affinity between Hemigasterini and Ichneumoninae was uncertain in the mapping analyses (Figure S9). When we ignored *Colocnema rufina* and *Acrolyta* sp. (Phygadeuontinae), the topologies from the AA matrix were all identical in Ichneumoniformes, (Agriotypinae + (Cryptinae + ((Adelognathinae + (Ateleutinae + Microleptinae)) + Ichneumoninae)).

Ophioniformes is the largest higher-level grouping that contains 12 certain subfamilies and the conflict subfamilies Lycorininae and Cremastinae (Figure 4). The members of higher Ophioniformes and lower Ophioniformes remain not fully clear. For higher Ophioniformes, the topology of (Anomaloninae + (Campopleginae, Nesomesochorinae, Ophioninae)) in higher Ophioniformes was fully supported. Nesomesochorinae was sister to Ophioninae or Campopleginae (Figure 4 and Figures S6–S9). Most phylogenetic analyses and conflict examination supported the finding that *Colpotrochia* sp. and *Triclistus* sp. was sister to these four certain subfamilies of higher Ophioniformes (Figure 4 and Figure 5C). Lycorininae and Cremastinae are grouped with full support and may be associated to higher Ophioniformes with support values of 72.9% (AA) and 67.0% (NU) in the likelihood mapping analyses (Figure 5B). The rearrangement events (the transposition of *trnL2*) also suggested that Hybrizontinae (R28) and Cremastinae may be included in the higher Ophioniformes. For lower Ophioniformes, Ctenopelmatinae was never recovered as a monophyletic. *Opheltes* sp. (Perilissini) and *Scolobates* sp. (Scolobatindi), as a monophyletic group, were far away from Ctenopelmatinae. However, the placement of *Netelia* spp. (Phytodietini, Tryphoninae) remained unclear, which either was sister to other Ophioniformes based on NU matrix or close to Tersilochinae with strong support from the AA matrix.

## 4. Discussion

### 4.1. Comparative Mitogenomics of Ichneumonidae

Ichneumonidae, as with other parasitoid wasps, have the higher A + T content and a negative GC skew, despite differing to the sister family Braconidae by a reversal of strand asymmetry on a positive GC skew (Figure 1) [34]. Because the base composition bias was also found in protein-coding gene (PCGs) sequences, we considered that the reversal in Braconidae was caused by codon usage bias rather than the common GC skew in Ichneumonidae. Subsequently, the more synonymous codon usage bias (SCUB) toward G nucleotides (e.g., the GGG coding Gln) in Braconidae was detected, rather than those in Ichneumonidae (Figure S10). Thus, we illustrated that the reversal of strand asymmetry [35,36] in the mitochondrial genomes of Braconidae is mainly caused by codon usage bias towards codons with G nucleotides. For Ichneumonidae, we suggested that the mutation is almost neutral, maintaining a common codon usage bias. Ichneumonidae and Braconidae were certainly different on RUSC (Figure 1F). We also found that the base composition on the first and second sites impacted largely on codon usage, more so than the third sites in Ichneumonidae (Figure S11). This implied that there was more non-synonymous (dN) mutation-impacted codon usage, even gene expression products, than in other families of parasitic Hymenoptera. The evolutionary rate of mitochondrial genomes are low, as reported elsewhere [32,33]. It is indicated that most mutations were synonymous or even purified by selection pressure.

These characteristics suggested that ichneumonid mitochondrial genes under low selection pressure may contain a good signal for phylogeny. Although the previous studies considered that the factors influencing the fast rate of gene rearrangement remain unclear in insect mitochondrial genomes [20,27,56], we suggested that the mitogenomes, such as those of the Ichneumonidae with low evolutionary rates and maintaining neutral mutation, can be used to explore phylogeny. Moreover, there are no extremely outstanding long branches and attractions in all phylogenetic trees, which confirmed the presence of homogeneous evolution with the extensive sampling of mitochondrial genomes within this family. It seems that features of the mitochondrial genome can provide information in phylogenetic inferences, but these features, whether related to the species diversity or the patterns of genome evolution, remain unclear.

The gene orders of mitochondrial genomes were diverse in Ichneumonidae. The most rearrangements which caused gene order variable occurred in or among tRNA gene clusters. However, other parasitoid wasps have more rearrangements of protein-coding genes, such as chalcidoids, sphecids and chrysidoids [25,28,29]. We found that R1 is widespread, and nearly all other types are based on R1. However, the rearrangement of Brachycyrtiformes seems not to be based on R1, which helps to confirm its placement. We also inferred that R1 evolved after the split of Brachycyrtiformes, according to the phylogenetic relationships. Ophioniformes, the high-level groupings with large diversification in both biology and morphology, have the most varied rearrangement types [6]. These rearrangement events can provide useful clues for subfamily relationships, as previously reported [25,28,29]. For example, the location of *trnL2* suggested that the subfamilies Hybrizontinae and Cremastinae are associated with higher Ophioniformes.

### 4.2. Phylogenetic Relationships within Ichneumonidae

We placed Eucerotinae in Brachycyrtiformes with strong support from all topologies and rearrangements, as did Santos et al. [16,17], although the placement of Eucerotinae is unclear in some studies [2,6,13], and recently, two studies suggest Brachycyrtiformes or only Eucerotinae are sisters to the Ichneumoniformes [15,17]. Our results strongly showed that another unplaced subfamily, Microleptinae, is sister to Ateleutinae in Ichneumoniformes, which is in agreement with recent studies [13,16,17]. Xoridiformes has been considered to be the sister lineage to the rest of Ichneumonidae [2,14,15,19], while Bennett et al. [13] disagreed, but did not reach a conclusion. We agreed that Xoridiformes was sister to ((Pimpliformes + Ichneumoniformes) + Ophioniformes), but Brachycyrtiformes was the sister to the rest of the ichneumonids, as mentioned above. Unfortunately, the recent molecular study with a large taxon or gene sampling had not included the key subfamily Brachycyrtinae [15], and our study did not include the rare subfamily Labeninae, either. The topology of ((Pimpliformes + Ichneumoniformes) + Ophioniformes) is uncontroversial with most studies [13,14,15,18]. There are some controversies within the three main high-level groupings from different analyses, but the better identities are put forward by the same matrices, rather than methods. We recommend the results from the AA matrix because of the above arguments (Figure 4).

Lycorininae and Cremastinae were grouped together by our inferences. We tend to include Lycorininae and Cremastinae in the higher Ophioniformes, as suggested by Quicke et al. [2] and Bennett et al. [13], based on the results from the *trnL2* transposition of Cremastinae and the mapping analyses. The heterogeneity analysis confirmed that they are a bit away from others (Figure S5), which might indicate their faster evolution, leading to the variable placements in our topologies. Additionally, another limit of this problem is the lack of samples of Cremastinae, which is a subfamily with more than 30 genera. Therefore, this problem needs to be resolved by including more genera from Cremastinae and relative lineages.

Hybrizontinae, the wasps who attack ants, was closer to other hymenopteran parasitoids, such as Mesochorinae and Ctenopelmatinae in lower Ophioniformes according to our analyses, and clustered with Mesochorinae, the hyperparasitoids of Ichneumonoidea, based on the AA matrix. We investigated codon and amino acid usage bias, and observed that Hybrizontinae was more different than other species in our study (Figure S7), and this may account for its various positions in different analyses. However, the rearrangement of R28 suggested that they are associated with higher Ophioniformes, as previous reported [6].

## 5. Conclusions

We found that the mitochondrial genomes of ichneumonid wasps were highly conserved in base composition and with low evolutionary rates, and were diverse in gene order. Furthermore, we confirmed that the bias of base composition and codon usage in Ichneumonidae was caused by natural mutation under a lower selection pressure than in Braconidae. The mitochondrial genomes of Ichneumonidae contain suitable signals for phylogenetic analysis. There are 38 types of gene order in 104 ichneumonid species, of which R1, with a shuffled tRNA cluster, *trnW-trnY-trnC* and *trnI-trnQ-trnM*, is commonly found in the high-level groupings, except for Brachycyrtiformes, while most other types of gene order are based on R1. It is obvious that the rearrangement events are more frequent in Ophioniformes than in Ichneumoniformes and Pimpliformes.

Finally, the most certain relationships within Ichneumonidae were reconstructed with the mitochondrial data. Phylogenetic topologies were constructed for 104 species, representing 33 ichneumonid subfamilies that formed five higher-level groupings: Brachycyrtiformes, Ichneumoniformes, Ophioniformes, Pimpliformes and Xoridiformes. The two formerly unplaced Eucerotinae and Microleptinae subfamilies were placed in Brachycyrtiformes and Ichneumoniformes with strong supports, respectively. Brachycyrtiformes was found to be the sister to the rest of the ichneumonids. Our results also strongly support the monophyletic group, Pimpliformes + Ichneumoniformes, which is sister to Ophioniformes. We suggest that rearrangement events can help to understand phylogenetic relationships, for example, the transposition of gene *trnL2* in higher Ophioniformes.

The taxa sampling (104 species) within Ichneumonidae in this study makes it one of the most comprehensive comparative mitochondrial genomics and phylogenetic studies of a family in Hymenoptera. We provided a new perspective and data support for the current ichneumonid phylogeny, which will help to understand the diversity and evolution of the Darwin wasps and provide good material for evolutionary biology.

## Data Availability

The new mitogenome assemblies and annotation data in this study have been submitted to the GenBank database under accession numbers in Table S1.

## Supplementary Materials

The following are available online at https://www.mdpi.com/article/10.3390/insects13020124/s1, Table S1: Information for the mitochondrial genomes used for phylogenetic inference. Table S2: Information for the mitochondrial genomes exclude Ichneumonidae used for comparative analysis. Table S3: Partitions and their best model list. Figure S1: The mitochondrial genes arrangement pattern of Ichneumonidae. Ther are 38 types of the rearrangement pattern (R1–R38). Figure S2: Base composition of the majority strand and the protein-coding genes (PCGs) of mitochondrial genomes for Ichneumonidae, Braconidae and other parasitoid Hymenoptera. Figure S3: The base substitutional saturation plots of 1st, 2nd and 3rd sites for 13 protein-coding genes (PCGs). Figure S4: Heterogeneity analysis for AA and NU matrixes. Figure S5: Ordination of 104 ichneumonid sequences on the two principal correspondence analysis axes. Figure S6: Phylogenetic relationships of Ichneumonid wasps inferred from amino acid sequences of 13 protein-coding genes in mitochondrial genomes (AA matrixes) by ML method. Figure S7: Phylogenetic relationships of Ichneumonid wasps inferred from nucleotide sequences of 13 protein-coding genes in mitochondrial genomes (NU matrixes) by BI inference. Figure S8: Phylogenetic relationships of Ichneumonid wasps inferred from nucleotide sequences of 13 protein-coding genes in mitochondrial genomes (NU matrixes) by ML method. Figure S9: Results from likelihood mapping. The analysis was based on both AA and NU matrixes. Figure S10: The mean for relative synonymous codon usage (RSCU) of 13 protein-coding genes in mitochondrial genomes of Ichneumonidae and Braconidae. Figure S11: The Correlation test for the factors that influence RSCU bias against the first major axis (Axis1/48.93% explanation of the variation) generated by Correspondence Analysis (COA) in Ichneumonidae, Braconidae and other parasitoid Hymenoptera.
