# Supplementary material for: Comparative Mitochondrial Genomics of 104 Darwin Wasps (Hymenoptera: Ichneumonidae) and Its Implication for Phylogeny
Source: Insects. 2022 Jan 25;13(2):124. doi: 10.3390/insects13020124 (PMC8874624; doi:10.3390/insects13020124)
Supplement: Supplementary file 1 [file insects-13-00124-s001.zip › insects-1549963-supplementary.pdf]

Supplementary Information

Comparative mitochondrial genomics of 104 Darwin wasps (Hymenoptera: Ichneumonidae) and its implication for phylogeny

Boying Zheng [1,2] Yuanyuan Han [1,2], Ruizhong Yuan [1,2], Jingxian Liu [3], Cornelis van Achterberg [1,2], Pu Tang [1,2,4,5,*] and Xuexin Chen [1,2,4,5]

1 State Key Laboratory of Rice Biology, Zhejiang University, Hangzhou 310058, China; zhengboyingg@163.com (B.Z.); yyhan6@zju.edu.cn (Y.H.); monareason@zju.edu.cn (R.Y.); kees@vanachterberg.org (C.A.); xxchen@zju.edu.cn (X.C.)
2 Institute of Insect Sciences, College of Agriculture and Biotechnology, Zhejiang University, Hangzhou 310058, China
3 Department of Entomology, College of Plant Protection, South China Agricultural University,
Guangzhou 510642, China; jingxianliu@foxmail.com
4 Ministry of Agriculture Key Lab of Molecular Biology of Crop Pathogens and Insects, Zhejiang University, Hangzhou 310058, China
5 Zhejiang Provincial Key Laboratory of Biology of Crop Pathogens and Insects, Zhejiang University,
Hangzhou 310058, China
* Correspondence: ptang@zju.edu.cn

Supplementary Contents

Supplementary Tables (S1- S3) **4**

Table S1 Information for the mitochondrial genomes used for phylogenetic inference. 4

Table S2 Information for the mitochondrial genomes exclude Ichneumonidae used for comparative analysis. 7

Table S3 Partitions and their best model list. 9

Supplementary Figures (S1- S11) **11**

Figure S1 The mitochondrial genes arrangement pattern of Ichneumonidae. Ther are 38 types of the rearrangement pattern (R1-R38). 12

Figure S2 Base composition of the majority strand and the protein-coding genes (PCGs) of mitochondrial genomes for Ichneumonidae, Braconidae and other parasitoid Hymenoptera. 13

Figure S3 The base substitutional saturation plots of 1st, 2nd and 3rd sites for 13 protein-coding genes (PCGs). 15

Figure S4 Heterogeneity analysis for AA and NU matrixes. 17

Figure S5 Ordination of 104 ichneumonid sequences on the two principal correspondence analysis axes. . 18

Figure S6 Phylogenetic relationships of Ichneumonid wasps inferred from amino acid sequences of 13 protein-coding genes in mitochondrial genomes (AA matrix) by ML method. 19

Figure S7 Phylogenetic relationships of Ichneumonid wasps inferred from nucleotide sequences of 13 protein-coding genes in mitochondrial genomes (NU matrix) by BI inference. 21

Figure S8 Phylogenetic relationships of Ichneumonid wasps inferred from nucleotide sequences of 13 protein-coding genes in mitochondrial genomes (NU matrix) by ML method. 22

Figure S9 Results from likelihood mapping. The analysis was based on both AA and NU matrixes. 26

Fig. S10 The mean for relative synonymous codon usage (RSCU) of 13 protein-coding genes in mitochondrial genomes of Ichneumonidae and Braconidae. 26

Figure S11 The Correlation test for the factors that influence RSCU bias against the first major axis (Axis1/48.93% explanation of the variation) generated by Correspondence Analysis (COA) in Ichneumonidae, Braconidae and other parasitoid Hymenoptera. 27

Supplementary Reference **28**

Supplementary Data

Supplementary Data S1 The general features of mitochondrial genomes of Ichneumonidae

Supplementary Data S2 Aligned Sequences files

Supplementary Tables (S1- S3)

Table S1 Information for the mitochondrial genomes used for phylogenetic inference.

Voucher Specimens	Fammily	Subfamily	Species	Collection Place	Accession Number	Reference
ZJUHW_202000001	Ichneumonidae	Rhyssinae	*Megarhyssa jezoensis*	Beijing, China	MT252834	This study
ZJUHW_202000002	Ichneumonidae	Rhyssinae	*Triancyra* sp.	Tibet, China	MT252835	This study
ZJUHW_202000003	Ichneumonidae	Anomaloninae	*Heteropelma* sp.	Qinghai, China	MT252836	This study
ZJUHW_202000004	Ichneumonidae	Anomaloninae	*Anomalon* sp.	Yunnan, China	MT252837	This study
ZJUHW_202000005	Ichneumonidae	Ctenopelmatinae	*Lagarotis* sp.	Hainan, China	MT252838	This study
ZJUHW_202000006	Ichneumonidae	Ctenopelmatinae	*Phobetes* sp.1	Liaoning, China	MT252839	This study
ZJUHW_202000007	Ichneumonidae	Ctenopelmatinae	*Phobetes* sp.2	Zhejiang, China	MT252840	This study
ZJUHW_202000008	Ichneumonidae	Ctenopelmatinae	*Opheltes* sp.	Guizhou, China	MT252841	This study
ZJUHW_202000009	Ichneumonidae	Ctenopelmatinae	*Pion* sp.	Guizhou, China	MT252842	This study
ZJUHW_202000010	Ichneumonidae	Ctenopelmatinae	*Scolobates* sp.	Hainan, China	MT252843	This study
ZJUHW_202000011	Ichneumonidae	Ctenopelmatinae	*Alexeter nebulator*	Henan, China	MT252844	This study
ZJUHW_202000012	Ichneumonidae	Banchinae	*Glyptopimpla* sp.	Tibet, China	MT252845	This study
ZJUHW_202000013	Ichneumonidae	Banchinae	*Orientoglypta* sp.1	Liaoning, China	MT252846	This study
ZJUHW_202000014	Ichneumonidae	Banchinae	*Orientoglypta* sp.2	Liaoning, China	MT252847	This study
ZJUHW_202000015	Ichneumonidae	Banchinae	*Lissonota* sp.	Zhejiang, China	MT252848	This study
ZJUHW_202000016	Ichneumonidae	Banchinae	*Glypta wahli*	Zhejiang, China	MT252849	This study
ZJUHW_202000017	Ichneumonidae	Xoridinae	*Odontocolon* sp.	Liaoning, China	MT252850	unpublished
ZJUHW_202000018	Ichneumonidae	Xoridinae	*Xorides funiuensis*	Sichuan, China	MT252851	unpublished
ZJUHW_202000019	Ichneumonidae	Eucerotinae	*Euceros kiushuensis*	Tibet, China	MT252852	unpublished
ZJUHW_202000020	Ichneumonidae	Eucerotinae	*Euceros serricornis*	Sichuan, China	MT252853	unpublished
ZJUHW_202000021	Ichneumonidae	Diplazontinae	*Promethes* sp.	Sichuan, China	MT252854	This study
ZJUHW_202000022	Ichneumonidae	Diplazontinae	*Syrphoctonus* sp.	Yunnan, China	MT252855	This study
ZJUHW_202000023	Ichneumonidae	Diplazontinae	*Sussaba sugiharai*	Yunnan, China	MT252856	This study
ZJUHW_202000024	Ichneumonidae	Diplazontinae	*Diplazon laetatorius*	Yunnan, China	MT252857	This study
ZJUHW_202000026	Ichneumonidae	Microleptinae	*Microleptes* sp.	Liaoning, China	MT252859	This study
ZJUHW_202000028	Ichneumonidae	Diacritinae	*Diacritus aciculatus*	Liaoning, China	MT252861	This study
ZJUHW_202000029	Ichneumonidae	Cylloceriinae	*Hyperacmus crassicornis*	Yunnan, China	MT252862	This study
ZJUHW_202000030	Ichneumonidae	Cylloceriinae	*Cylloceria* sp.	Liaoning, China	MT252863	This study
ZJUHW_202000031	Ichneumonidae	Ophioninae	*Ophion* sp.	Liaoning, China	MT252864	This study
ZJUHW_202000032	Ichneumonidae	Ophioninae	*Enicospilus* sp.2	Tibet, China	MT252865	This study
ZJUHW_202000033	Ichneumonidae	Agriotypinae	*Agriotypus* sp.	Zhejiang, China	MT252866	This study
ZJUHW_202000034	Ichneumonidae	Hybrizontinae	*Hybrizon hei*	Shandong, China	MT252867	This study
ZJUHW_202000035	Ichneumonidae	Hybrizontinae	*Hybrizon xu*	Shandong, China	MT252868	This study
ZJUHW_202000036	Ichneumonidae	Poemeniinae	*Eugalta* sp.1	Sichuan, China	MT252869	This study
ZJUHW_202000037	Ichneumonidae	Poemeniinae	*Cnastis* sp.	Guizhou, China	MT252870	This study

ZJUHW_202000038	Ichneumonidae	Poemeniinae	*Eugalta* sp.2	Taiwan, China	MT252871	This study
ZJUHW_202000039	Ichneumonidae	Cryptinae	*Acroricnus chinensis*	Yunnan, China	MT302538	This study
ZJUHW_202000040	Ichneumonidae	Cryptinae	*Gambrus* sp.	Tibet, China	MT252872	This study
ZJUHW_202000041	Ichneumonidae	Cryptinae	*Kemalia* sp.	Jiangxi, China	MT252873	This study
ZJUHW_202000042	Ichneumonidae	Cryptinae	*Friona* sp.	Jiangxi, China	MT252874	This study
ZJUHW_202000043	Ichneumonidae	Cryptinae	*Dagathia* sp.	Liaoning, China	MT252875	This study
ZJUHW_202000044	Ichneumonidae	Phygadeuontinae	*Colocnema rufina*	Shaanxi, China	MT252876	This study
ZJUHW_202000045	Ichneumonidae	Ateleutinae	*Ateleute* sp.	Yunnan, China	MT252877	This study
ZJUHW_202000046	Ichneumonidae	Pimplinae	*Pimpla aequalis*	Zhejiang, China	MT252878	This study
ZJUHW_202000047	Ichneumonidae	Pimplinae	*Acropimpla* sp.	Zhejiang, China	MT252879	This study
ZJUHW_202000048	Ichneumonidae	Pimplinae	*Scambus* sp.1	Yunnan, China	MT252880	This study
ZJUHW_202000049	Ichneumonidae	Pimplinae	*Sinarachna* pallipes	Yunnan, China	MT252881	This study
ZJUHW_202000050	Ichneumonidae	Pimplinae	*Scambus* sp.2	Yunnan, China	MT252882	This study
ZJUHW_202000051	Ichneumonidae	Pimplinae	*Dolichomitus irritator*	Yunnan, China	MT252883	This study
ZJUHW_202000052	Ichneumonidae	Mesochorinae	*Mesochorus* sp.1	Jiangxi, China	MT252884	This study
ZJUHW_202000053	Ichneumonidae	Mesochorinae	*Mesochorus* sp.2	Jiangxi, China	MT252885	This study
ZJUHW_202000054	Ichneumonidae	Mesochorinae	*Mesochorus* sp.3	Henan, China	MT252886	This study
ZJUHW_202000055	Ichneumonidae	Mesochorinae	*Mesochorus testaceus*	Yunnan, China	MT252887	This study
ZJUHW_202000056	Ichneumonidae	Acaenitinae	*Yamatarotes bicolor*	Liaoning, China	MT252888	This study
ZJUHW_202000057	Ichneumonidae	Acaenitinae	*Jezarotes* sp.1	Yunnan, China	MT252889	This study
ZJUHW_202000058	Ichneumonidae	Acaenitinae	*Jezarotes* sp.2	Jilin, China	MT252890	This study
ZJUHW_202000059	Ichneumonidae	Collyriinae	*Bicurta* sp.	Liaoning, China	MT252891	This study
ZJUHW_202000060	Ichneumonidae	Ichneumoninae	*Coelichneumon bivittatus*	Zhejiang, China	MT302534	This study
ZJUHW_202000061	Ichneumonidae	Ichneumoninae	*Vulgichneumon saevus*	Tibet, China	MT252892	This study
ZJUHW_202000062	Ichneumonidae	Ichneumoninae	*Listrodromus* sp.	Tibet, China	MT252893	This study
ZJUHW_202000063	Ichneumonidae	Ichneumoninae	*Oronotus* sp.	Jiangxi, China	MT252894	This study
ZJUHW_202000064	Ichneumonidae	Lycorininae	*Lycorina triangulifera*	Heilongjiang, China	MT252895	This study
ZJUHW_202000065	Ichneumonidae	Lycorininae	*Lycorina* sp.	Shandong, China	MT252896	This study
ZJUHW_202000066	Ichneumonidae	Adelognathinae	*Adelognathus* sp.	Sichuan, China	MT252897	This study
ZJUHW_202000067	Ichneumonidae	Orthocentrinae	*Stenomacrus* sp.	Sichuan, China	MT252898	This study
ZJUHW_202000068	Ichneumonidae	Orthocentrinae	*Plectiscus* sp.1	Zhejiang, China	MT252899	This study
ZJUHW_202000069	Ichneumonidae	Orthocentrinae	*Orthocentrus* sp.	Zhejiang, China	MT252900	This study
ZJUHW_202000070	Ichneumonidae	Orthocentrinae	*Plectiscus* sp.3	Zhejiang, China	MT252901	This study
ZJUHW_202000071	Ichneumonidae	Orthocentrinae	*Plectiscidea* sp.	Zhejiang, China	MT252902	This study
ZJUHW_202000072	Ichneumonidae	Orthocentrinae	*Plectiscus* sp.2	Zhejiang, China	MT252903	This study
ZJUHW_202000073	Ichneumonidae	Orthocentrinae	*Proclitus* sp.	Zhejiang, China	MT252904	This study
ZJUHW_202000074	Ichneumonidae	Cremastinae	*Trathala* sp.1	Liaoning, China	MT252905	This study
ZJUHW_202000075	Ichneumonidae	Cremastinae	*Trathala* sp.2	Yunnan, China	MT252906	This study
ZJUHW_202000076	Ichneumonidae	Metopiinae	*Colpotrochia* sp.	Tibet, China	MT252907	This study
ZJUHW_202000077	Ichneumonidae	Metopiinae	*Hypsicera* sp.3	Zhejiang, China	MT252908	This study

ZJUHW_202000078	Ichneumonidae	Metopiinae	*Chorinaeus* facilis	Guangdong, China	MT252909	This study
ZJUHW_202000079	Ichneumonidae	Metopiinae	*Triclistus* sp.	Guangdong, China	MT252910	This study
ZJUHW_202000080	Ichneumonidae	Metopiinae	*Exochus* sp.	Guangdong, China	MT252911	This study
ZJUHW_202000081	Ichneumonidae	Metopiinae	*Hypsicera* sp.2	Guangdong, China	MT252912	This study
ZJUHW_202000082	Ichneumonidae	Metopiinae	*Metopius* sp.	Henan, China	MT252913	This study
ZJUHW_202000083	Ichneumonidae	Tersilochinae	*Phradis* sp.	Tibet, China	MT252914	This study
ZJUHW_202000084	Ichneumonidae	Tersilochinae	*Probles* sp.1	Sichuan, China	MT252915	This study
ZJUHW_202000085	Ichneumonidae	Tersilochinae	*Probles* sp.2	Gansu, China	MT302535	This study
ZJUHW_202000086	Ichneumonidae	Brachycyrtinae	*Brachycyrtus ornatus*	Liaoning, China	MT252916	This study
ZJUHW_202000087	Ichneumonidae	Tryphoninae	*Netelia* sp.1	Hainan, China	MT252917	This study
ZJUHW_202000088	Ichneumonidae	Tryphoninae	*Netelia* sp.2	Liaoning, China	MT252918	This study
ZJUHW_202000089	Ichneumonidae	Tryphoninae	*Eridolius* sp.	Zhejiang, China	MT252919	This study
ZJUHW_202000090	Ichneumonidae	Tryphoninae	*Dyspetes luteomarginatus*	Sichuan, China	MT252920	This study
ZJUHW_202000091	Ichneumonidae	Tryphoninae	*Atopotrophos bucephalus*	Liaoning, China	MT252921	This study
ZJUHW_202000092	Ichneumonidae	Tryphoninae	*Oedemopsis* sp.	Zhejiang, China	MT252922	This study
ZJUHW_202000093	Ichneumonidae	Oxytorinae	*Oxytorus luridator*	Zhejiang, China	MT252923	This study
ZJUHW_202000094	Ichneumonidae	Nesomesochorinae	*Klutiana* sp.	Jilin, China	MT252924	This study
ZJUHW_202000095	Ichneumonidae	Pimplinae	*Theronia laevigata*	Liaoning, China	MT252925	This study
ZJUHW_202000096	Ichneumonidae	Diplazontinae	*Syrphoctonus nigritarsus*	Sichuan, China	MT252926	This study
ZJUHW_202000097	Ichneumonidae	Cryptinae	*Hidryta fusiventris*	Liaoning, China	MT302536	This study
ZJUHW_202000098	Ichneumonidae	Phygadeuontinae	*Acrolyta* sp.	Liaoning, China	MT302537	This study
——	Ichneumonidae	Ichneumoninae	*Amblyjoppa* sp.	——	MG923483	Tang et al. [1]
——	Aulacidae	——	*Aulacus sinensis*	——	MG923485	Tang et al. [1]
——	Braconidae	Microgastrinae	*Cotesia vestalis*	——	FJ154897	Wei et al. [2]
——	Ichneumonidae	Campopleginae	*Diadegma semiclausum*	——	EU871947	Wei et al. [3]
——	Ichneumonidae	Ichneumoninae	*Diadromus collaris*	——	JX131613	Li et al. [4]
——	Ichneumonidae	Ophioninae	*Enicospilus* sp.1	——	FJ478177	Dowton et al. [5]
——	Gasteruptiidae	——	*Gasteruption* sp.	——	KJ619460	Mao et al. [6]
——	Ichneumonidae	Campopleginae	*Hyposoter* sp.	——	MG923499	Tang et al. [1]
——	Ichneumonidae	Metopiinae	*Hypsicera* sp.1	——	MG923500	Tang et al. [1]
——	Trigonalidae	——	*Orthogonalys pulchella*	——	KJ619461	Mao et al. [6]
——	Ichneumonidae	Pimplinae	*Pimpla luctuosa*	——	MG923506	Tang et al. [1]
——	Aulacidae	——	*Pristaulacus compressus*	——	KF500406	Wei et al. [7]
——	Braconidae	Doryctinae	*Spathius agrili*	——	FJ387020	Wei et al. [2]
——	Trigonalidae	——	*Taeniogonalos taihorina*	——	KJ684986	Wu et al. [8]
——	Ichneumonidae	Campopleginae	*Venturia canescens*	——	FJ478176	Dowton et al. [5]
——	Braconidae	Meteorinae	*Zele chlorophthalmus*	——	MG822749	Direct Submission

Note: "——" represent that information is unknown or null for subfamilies.

Table S2 Information for the mitochondrial genomes exclude Ichneumonidae used for comparative analysis.

Sequence Name	Superfamily	Family	Accession Number	Reference
Alloxysta sp.	Cynipoidea	Figitidae	MG923482	Tang et al. [1]
Aulacus sinensis	Evanioidea	Aulacidae	MG923485	Tang et al. [1]
Belyta sp.	Diaprioidea	Diapriidae	MG923486	Tang et al. [1]
Brachymeria sp.	Chalcidoidea	Chalcididae	MG923487	Tang et al. [1]
Ceraphron sp.	Ceraphronoidea	Ceraphronidae	KJ570858	Mao et al. [6]
Ceraphronidae sp.	Ceraphronoidea	Ceraphronidae	MG923488	Tang et al. [1]
Ceratobaeus sp.	Platygastroidea	Platygastridae	KF696669	Mao and Dowton [9]
Conostigmus sp.	Ceraphronoidea	Megaspilidae	KF015227	Mao et al. [10]
Cotesia vestalis	Ichneumonoidea	Braconidae	FJ154897	Wei et al. [2]
Dendrocerus sp.	Ceraphronoidea	Megaspilidae	MG923490	Tang et al. [1]
Diapriidae sp.	Diaprioidea	Diapriidae	MG923491	Tang et al. [1]
Ettchellsia sinica	Megalyroidea	Megalyridae	KR270641	Tang et al. [1]
Eupelmus sp.	Chalcidoidea	Eupelmidae	MG923493	Tang et al. [1]
Eurytoma sp.	Chalcidoidea	Eurytomidae	MG923494	Tang et al. [1]
Evania appendigaster	Evanioidea	Evanidae	FJ593187	Wei et al. [11]
Exallonyx sp.	Proctotrupoidea	Proctotrupidae	MG923495	Tang et al. [1]
Foenatopus ruficollis	Stephanoidea	Stephanidae	KR270642	Tang et al. [1]
Gasteruption parvicollarium	Evanioidea	Gasteruptiidae	KR270643	Tang et al. [1]
Gasteruption sp.	Evanioidea	Gasteruptiidae	KJ619460	Mao et al. [10]
Gasteruption tournieri	Evanioidea	Gasteruptiidae	MG923496	Tang et al. [1]
Gastraspis sp.	Cynipoidea	Figitidae	MG923497	Tang et al. [1]
Helorus sp.	Proctotrupoidea	Heloridae	MG923498	Tang et al. [1]
Ibalia leucospoides	Cynipoidea	Ibaliidae	KJ814197	Mao et al. [12]
Idris sp.	Platygastroidea	Platygastridae	KF696670	Mao and Dowton [9]
Ismarus sp.	Diaprioidea	Diapriidae	MG923501	Tang et al. [1]
Leptopilina boulardi	Cynipoidea	Figitidae	KU665622	Oliveira et al. [13]
Megalyra sp.	Megalyroidea	Megalyridae	KJ577600	Mao et al. [6]
Megaphragma amalphitanum	Chalcidoidea	Trichogrammatidae	KT373787	Direct Submission
Megaspilidae sp.	Ceraphronoidea	Megaspilidae	MG923503	Tang et al. [1]
Monomachus antipodalis	Diaprioidea	Monomachidae	KM104168	Mao et al. [12]
Orthogonalys pulchella	Trigonalyoidea	Trigonalyidae	KJ619461	Mao et al. [6]
Pelecinus polyturator	Proctotrupoidea	Pelecinidae	KM104167	Mao et al. [12]
Philotrypesis pilosa	Chalcidoidea	Agaonidae	JF808723	Xiao et al. [14]

Platygastridae sp1	Platygastroidea	Platygastridae	MG923508	Tang et al. [1]
Platygastridae sp2	Platygastroidea	Platygastridae	MG923509	Tang et al. [1]
Platygastridae sp3	Platygastroidea	Platygastridae	MG923510	Tang et al. [1]
Pristaulacus compressus	Evanioidea	Aulacidae	NC022849	Wei et al. [7]
Prosevania sp.	Evanioidea	Evanidae	MG923512	Tang et al. [1]
Pteromalus puparum	Chalcidoidea	Pteromalidae	MG923513	Tang et al. [1]
Schlettererius cinctipes	Stephanoidea	Stephanidae	FJ478175	Dowton et al. [15]
Synergus sp.	Cynipoidea	Cynipidae	MG923514	Tang et al. [1]
Taeniogonalos taihorina	Trigonalyoidea	Trigonalyidae	KJ684986	Wu et al. [8]
Taeniogonalos tricolor	Trigonalyoidea	Trigonalyidae	MG923515	Tang et al. [1]
Telenomus_dignus	Platygastroidea	Scelionidae	KR270640	Direct Submission
Trissolcus basalis	Platygastroidea	Scelionidae	JN903532	Mao et al. [16]
Vanhornia eucnemidarum	Proctotrupoidea	Vanhorniidae	DQ302100	Castro et al. [17]
Acanthormius sp.	Ichneumonoidea	Braconidae	KF385867	Li et al. [18]
Afrocampsis griseosetosus	Ichneumonoidea	Braconidae	KJ412474	Li et al. [18]
Aphidius gifuensis	Ichneumonoidea	Braconidae	GU097658	Wei et al. [2]
Capitonius sp.	Ichneumonoidea	Braconidae	KF385869	Li et al. [18]
Cardiochiles fuscipennis	Ichneumonoidea	Braconidae	KF385870	Li et al. [18]
Cotesia vestalis	Ichneumonoidea	Braconidae	FJ154897	Wei et al. [2]
Diachasmimorpha longicaudata	Ichneumonoidea	Braconidae	GU097655	Wei et al. [2]
Elasmosoma sp.	Ichneumonoidea	Braconidae	KJ412470	Li et al. [18]
Eumacrocentrus sp.	Ichneumonoidea	Braconidae	KF385872	Li et al. [18]
Euurobracon breviterebrae	Ichneumonoidea	Braconidae	KF385871	Li et al. [18]
Histeromerus sp.	Ichneumonoidea	Braconidae	KF418765	Li et al. [18]
Homolobus sp.	Ichneumonoidea	Braconidae	KF385873	Li et al. [18]
Ichneutes sp.	Ichneumonoidea	Braconidae	KF385874	Li et al. [18]
Macrocentrus camphoraphilus	Ichneumonoidea	Braconidae	GU097656	Wei et al. [2]
Meteorus pulchricornis	Ichneumonoidea	Braconidae	GU097657	Wei et al. [2]
Mirax sp.	Ichneumonoidea	Braconidae	KJ412471	Li et al. [18]
Pambolus sp.	Ichneumonoidea	Braconidae	KF385875	Li et al. [18]
Paroligoneurus sp.	Ichneumonoidea	Braconidae	KJ412472	Li et al. [18]
Phanerotoma flava	Ichneumonoidea	Braconidae	GU097654	Wei et al. [2]
Proterops sp.	Ichneumonoidea	Braconidae	KJ412477	Li et al. [18]
Pselaphanus sp.	Ichneumonoidea	Braconidae	KF385876	Li et al. [18]
Pseudognaptodon sp.	Ichneumonoidea	Braconidae	KJ412473	Li et al. [18]
Sigalphus bicolor	Ichneumonoidea	Braconidae	KF385878	Li et al. [18]
Spathius agrili	Ichneumonoidea	Braconidae	FJ387020	Wei et al. [2]
Therophlius festivus	Ichneumonoidea	Braconidae	KF385868	Li et al. [18]

Triraphis sp.	Ichneumonoidea	Braconidae	KF385877	Li et al. [18]
Zele chlorophthalmus	Ichneumonoidea	Braconidae	MG822749	Direct Submission

Table S3 Partitions and their best model list.

Types	Partition Names	Gene of Sequences	Sites	Best Model from Modelfinder
AA	c1	Cytochrome oxidase subunit 1	1951-2475	mtART+F+R7
AA	c2	Cytochrome oxidase subunit 2	1721-1950	mtART+F+R6
AA	cb	Cytochrome b	3052-3425	mtART+F+R6
AA	a6	ATP synthase protein 6	2476-2693	mtART+F+R6
AA	n3	NADH dehydrogenase subunit 3	2694-2796	mtMet+F+I
AA	n2	NADH dehydrogenase subunit 2	2797-3051	mtZOA+F+R6
AA	a8	ATP synthase protein 8	792-828	mtZOA+F+I
AA	c3	Cytochrome oxidase subunit 3	524-791	mtART+F+R6
AA	n6	NADH dehydrogenase subunit 6	404-523	mtMet+F+R5
AA	n4	NADH dehydrogenase subunit 4	1-403	mtInv+F+R7
AA	n4l	NADH dehydrogenase subunit 4L	1632-1720	mtInv+F+G4
AA	n5	NADH dehydrogenase subunit 5	829-1320	mtInv+F+R8
AA	n1	NADH dehydrogenase subunit 1	1321-1631	mtZOA+F+R7
NU	n3p2	NADH dehydrogenase subunit 3	8024-8165	TVM+F+I+G4
NU	a6p3	ATP synthase protein 6	8513-8766	TIM2+F+R6
NU	n2p2	NADH dehydrogenase subunit 2	8767-9187	GTR+F+R4
NU	c3p1	Cytochrome oxidase subunit 3	8166-8512	GTR+F+R5
NU	n4lp2	NADH dehydrogenase subunit 4L	7453-7559	TVM+F+G4:
NU	n4p3	NADH dehydrogenase subunit 4	7560-8023	TIM+F+R6
NU	c1p3	Cytochrome oxidase subunit 1	6309-6802	TPM2+F+R7
NU	n2p1	NADH dehydrogenase subunit 2	6803-7223	K3Pu+F+R4
NU	cbp1	Cytochrome b	5938-6308	GTR+F+R5
NU	c2p2	Cytochrome oxidase subunit 2	7224-7452	GTR+F+R4
NU	c3p3	Cytochrome oxidase subunit 3	5591-5937	TIM2+F+R6
NU	c2p1	Cytochrome oxidase subunit 2	11967-12195	GTR+F+I+G4
NU	n6p1	ATP synthase protein 6	11716-11966	GTR+F+R5
NU	n5p3	NADH dehydrogenase subunit 5	11084-11715	TVM+F+R6
NU	a8p3	ATP synthase protein 8	11015-11083	K3Pu+F+R3

NU	n3p1	NADH dehydrogenase subunit 3	10873-11014	GTR+F+I+G4
NU	n5p1	NADH dehydrogenase subunit 5	10241-10872	GTR+F+R6
NU	n5p2	NADH dehydrogenase subunit 5	9609-10240	GTR+F+I+G4
NU	n2p3	NADH dehydrogenase subunit 2	9188-9608	K3Pu+F+R5
NU	cbp3	Cytochrome b	2631-3001	TN+F+R6
NU	n4p1	NADH dehydrogenase subunit 4	1938-2401	GTR+F+I+G4
NU	c2p3	Cytochrome oxidase subunit 2	2402-2630	TIM2+F+R5
NU	cbp2	Cytochrome b	720-1090	GTR+F+R5
NU	a8p2	ATP synthase protein 8	1091-1159	TIM3+F+R4
NU	a6p1	ATP synthase protein 6	359-612	TIM+F+R5
NU	n4lp3	NADH dehydrogenase subunit 4L	613-719	TIM3+F+G4:
NU	c1p1	Cytochrome oxidase subunit 1	1160-1653	GTR+F+I+G4
NU	n1p1	NADH dehydrogenase subunit 1	1654-1937	GTR+F+R6
NU	n4lp1	NADH dehydrogenase subunit 4L	1-107	TVM+F+G4:
NU	n6p2	NADH dehydrogenase subunit 6	108-358	GTR+F+I+G4
NU	n1p3	NADH dehydrogenase subunit 1	4813-5096	HKY+F+R6
NU	c1p2	Cytochrome oxidase subunit 1	5097-5590	TVM+F+R5
NU	n6p3	NADH dehydrogenase subunit 6	3682-3932	TN+F+R4
NU	c3p2	Cytochrome oxidase subunit 3	3933-4279	GTR+F+R6
NU	a8p1	ATP synthase protein 8	4280-4348	TPM2+F+R4
NU	n4p2	NADH dehydrogenase subunit 4	4349-4812	GTR+F+I+G4
NU	n1p2	NADH dehydrogenase subunit 1	3256-3539	GTR+F+I+G4
NU	n3p3	NADH dehydrogenase subunit 3	3540-3681	TIM2+F+R5
NU	a6p2	ATP synthase protein 6	3002-3255	TVM+F+I+G4

Note: Best models of 39 nucleic acids partitions (NU, every protein-coding gene was part to tree partitions according coding sites of p1, p2 and p3) and 13 amino acids partitions (AA) of 13 protein-coding genes in mitochondrial genomes from ModelFinder.

Supplementary Figures (S1- S11)

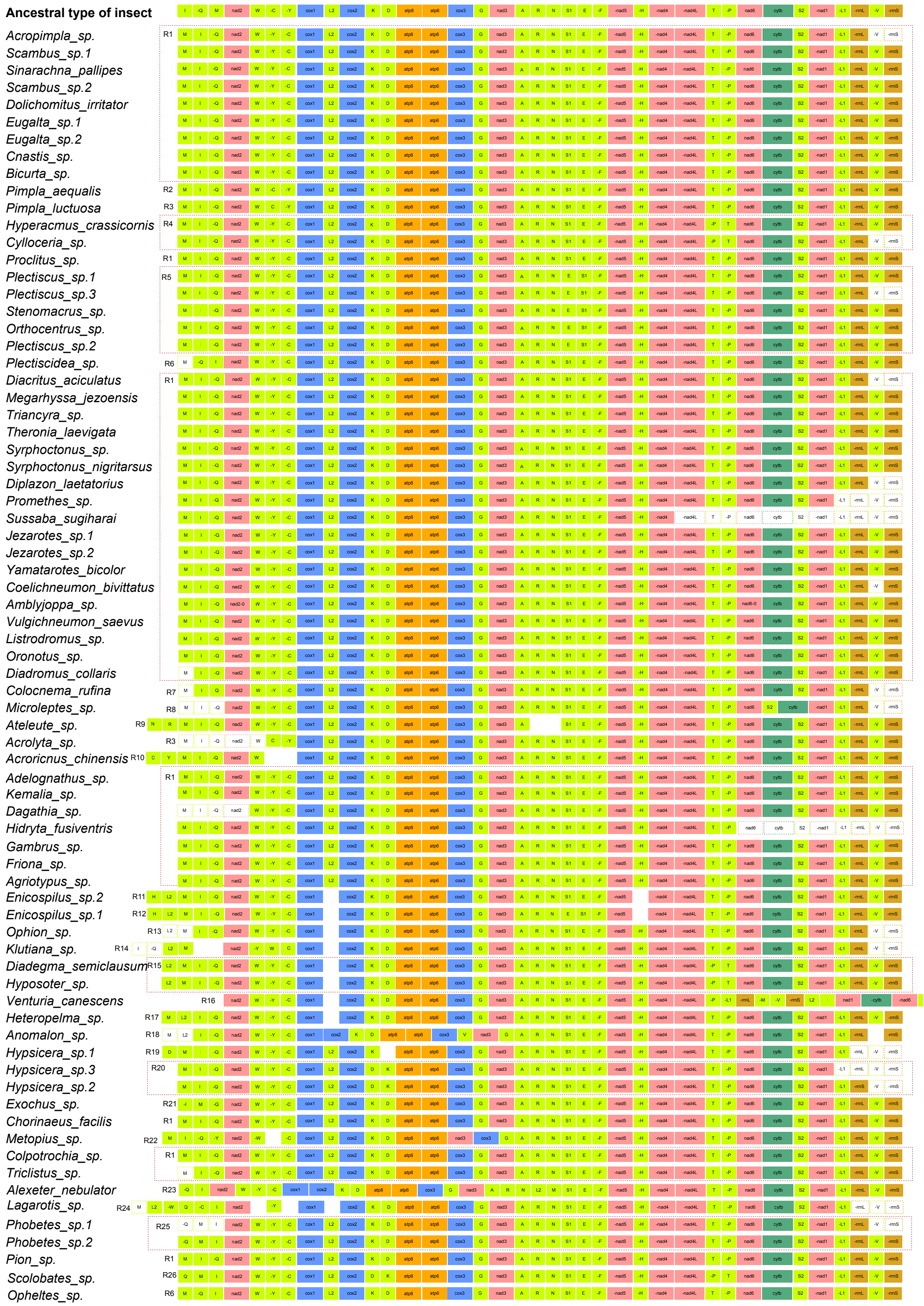

continued

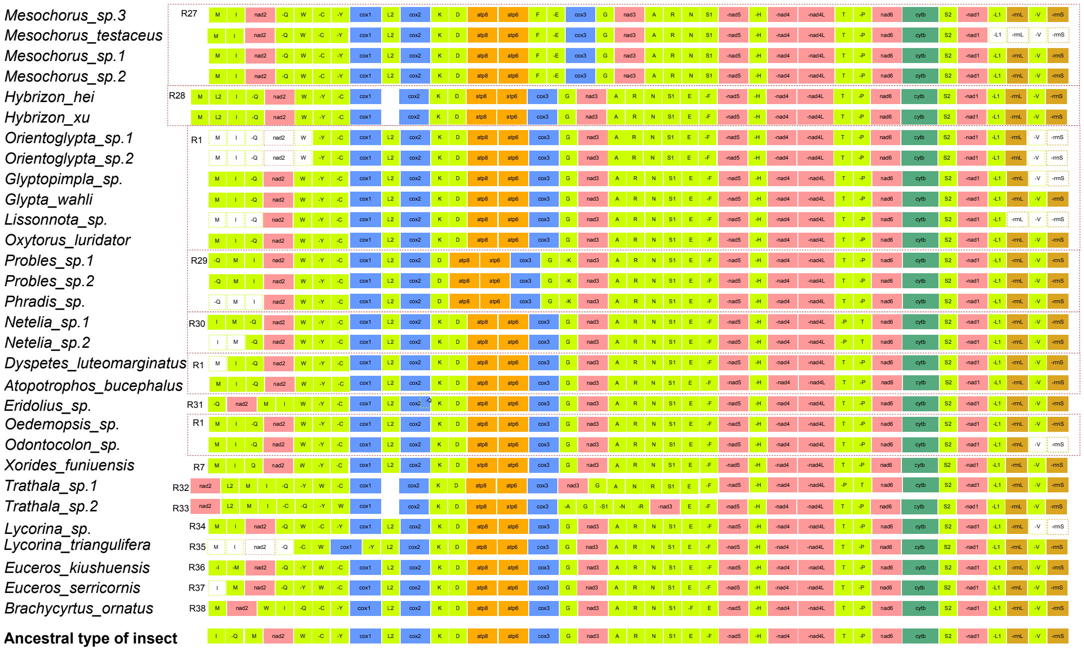

Figure S1 The mitochondrial genes arrangement pattern of Ichneumonidae. Ther are 38 types of the rearrangement pattern (R1-R38).

Lines in front of gene names indicate the gene coding on the minority strand. The genes sequenced failed are filled by blank blocks. The cox1- cox3: cytochrome oxidase subunits; cob: cytochrome b; nad1-nad6: NADH dehydrogenase com- ponents; rrnL and rrnS: ribosomal RNAs. Single letters identify the transfer RNA genes – refer to the IPUC-IUB (International Union of Pure and Applied Chemistry-International Union of Biochemistry) single-letter amino acid codes.

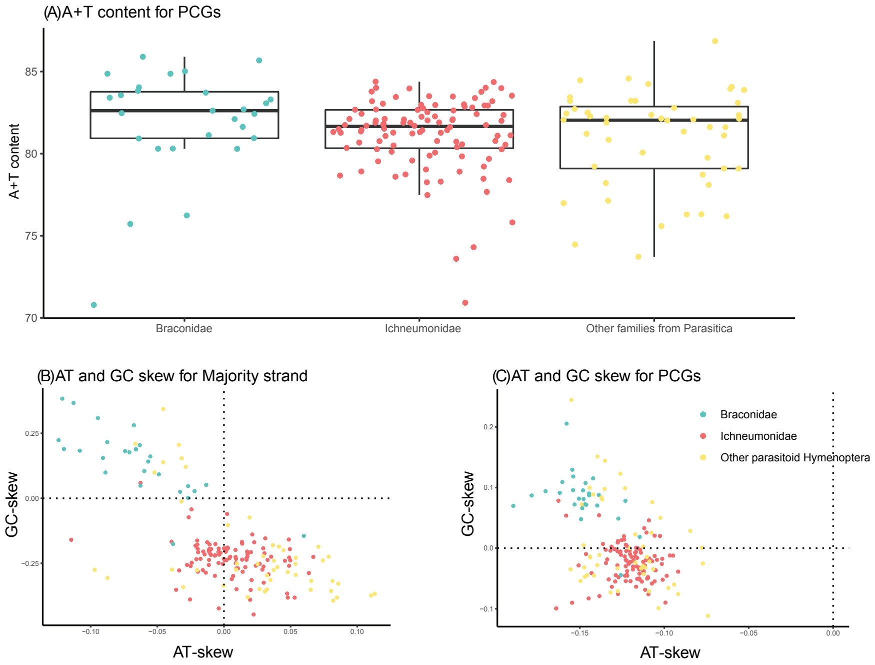

Figure S2 Base composition of the majority strand and the protein-coding genes (PCGs) of mitochondrial genomes for Ichneumonidae, Braconidae and other parasitoid Hymenoptera. (A) A + T content for the PCGs. (B) GC-skew against AT-skew of the majority strand. (C) GC-skew against AT-skew of PCGs. Each species is indicated by a filled circle.

The ts against tv on 1st sites of PCG

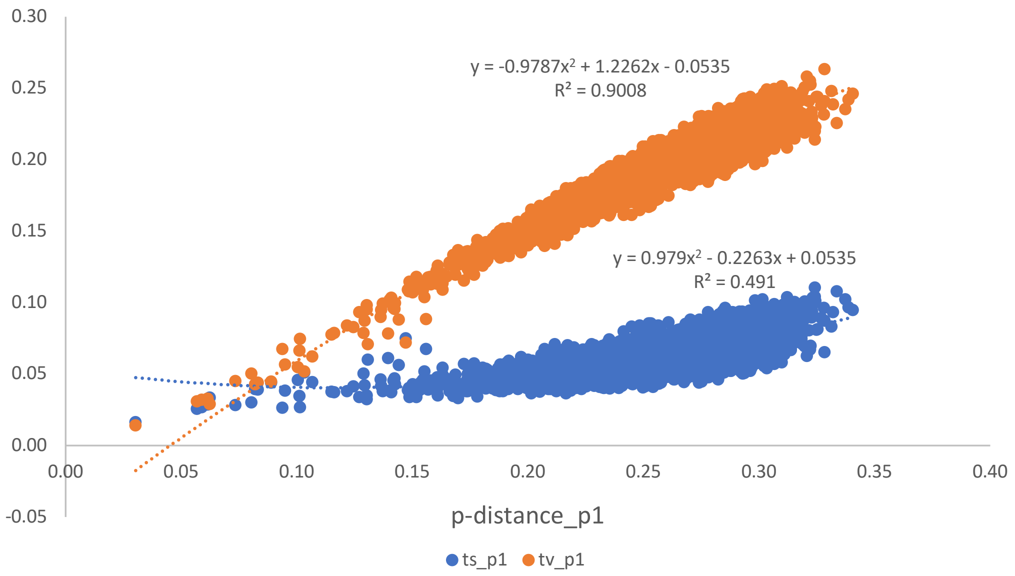

The ts against tv on 2nd sites of PCG

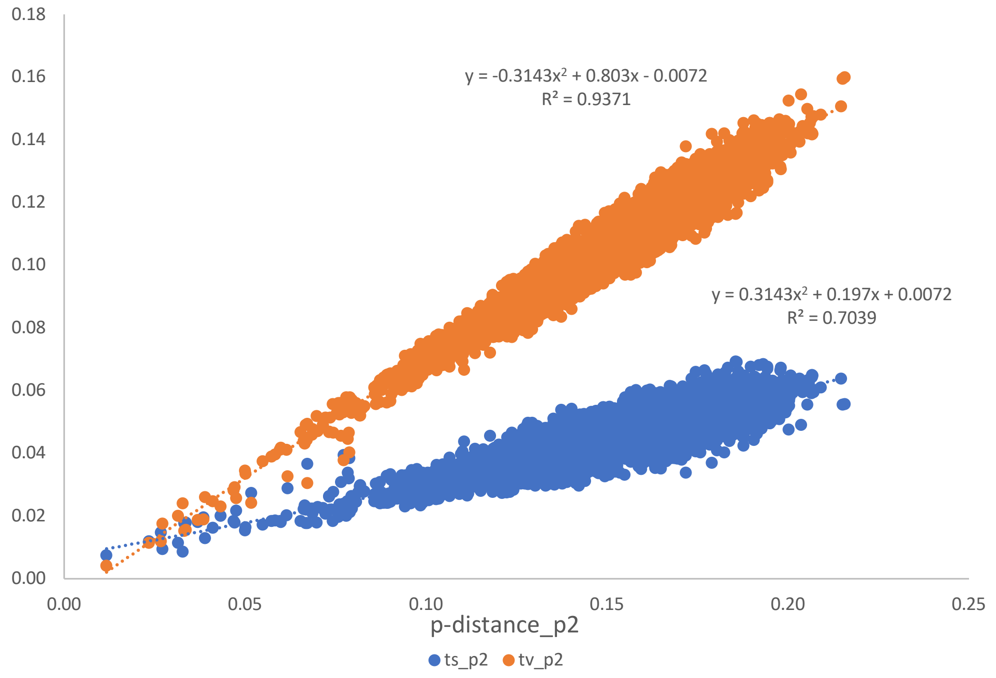

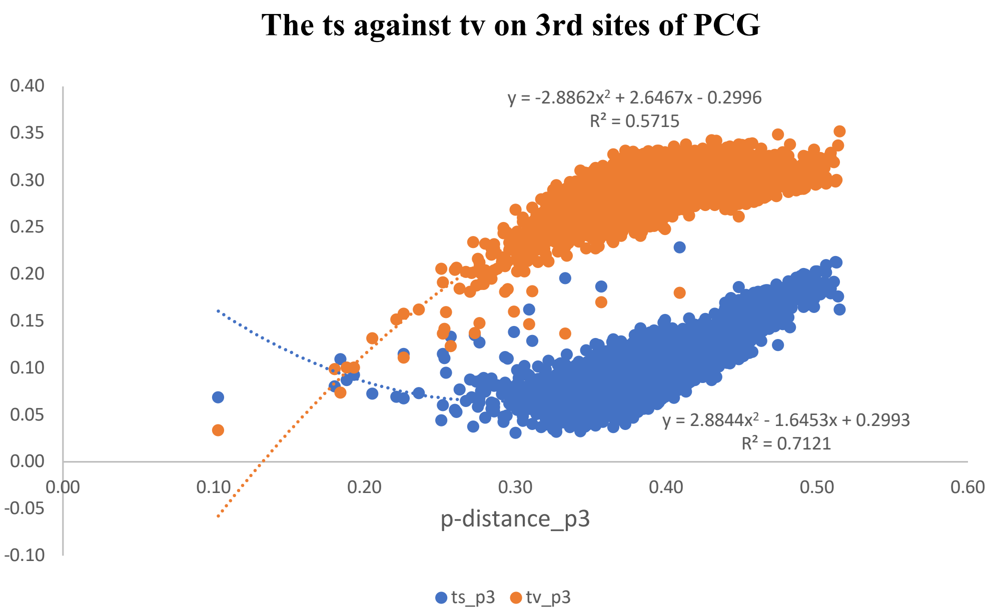

Figure S3 The base substitutional saturation plots of 1st, 2nd and 3rd sites for 13 protein-coding genes (PCGs).

The transitions (ts, blue) and transversions (tv, orange) against p- distance of both them, respectively.

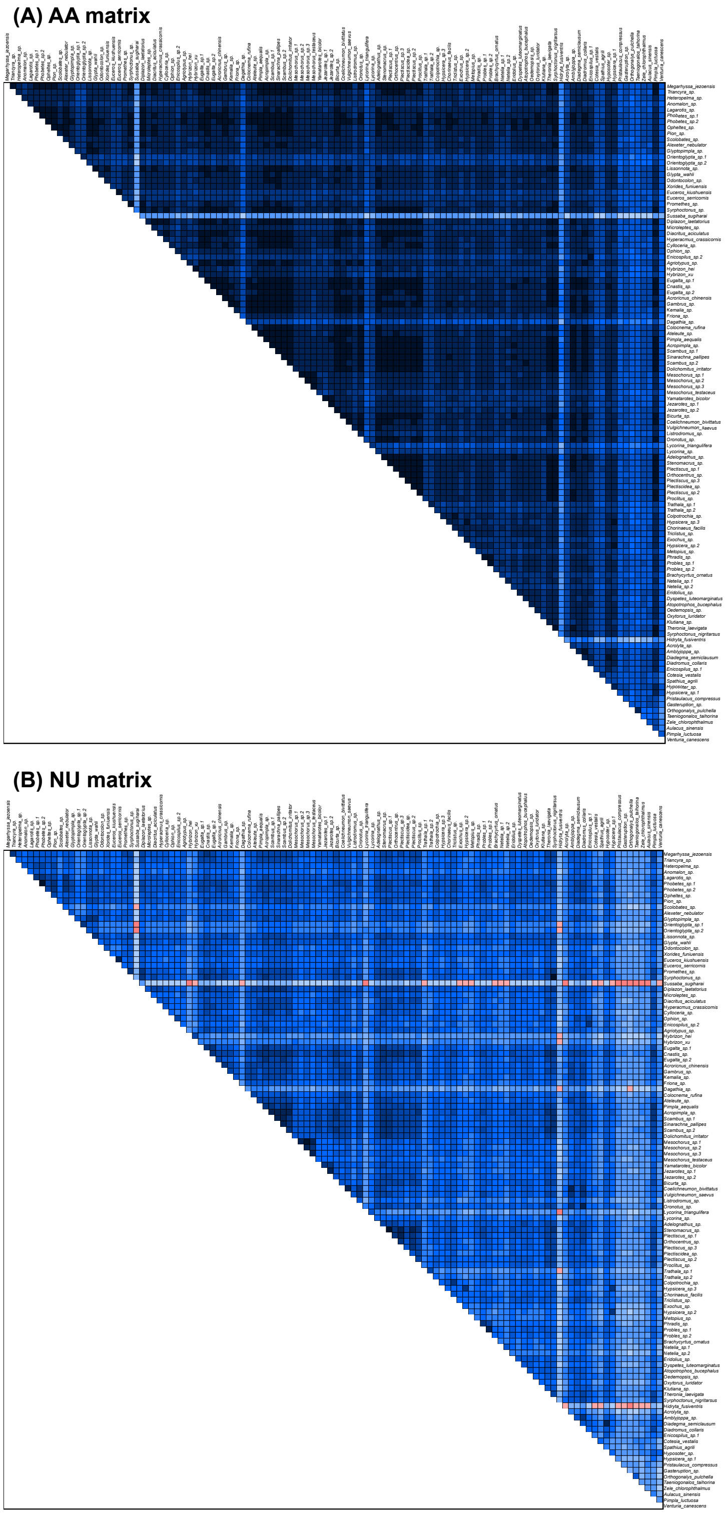

Figure S4 Heterogeneity analysis for AA and NU matrixes.

The mean similarity score between pairs of sequences is represented by a colored square, the red indicating great difference in sequence composition compared with the remainder and the blue indicating high similarity.

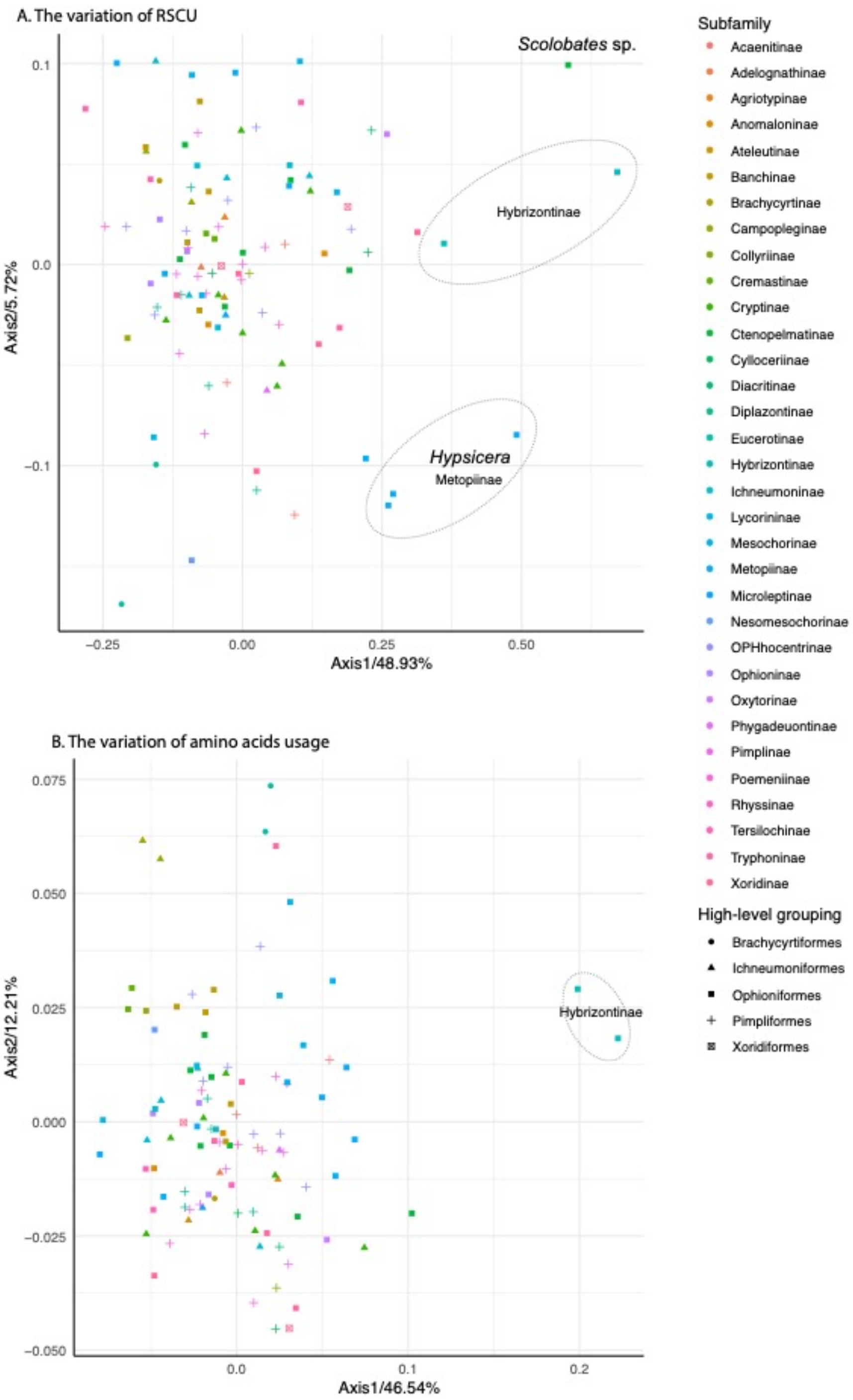

Figure S5 Ordination of 104 ichneumonid sequences on the two principal correspondence analysis axes.

The subfamilies are marked by different colors and the high-level groupings by shapes. The abnormal species are demonstrated by its position on Axis1 (circled and labeled). (A) There are 61 axes generated by CodonW using the codon usage of the 61 synonymous codonsamino acids. The total inertia of the RSCU is 0.055423. The explanation of the variation of Axis1 is 48.93% and Axis2 is 5.72%, others lower than 5%. (B) There are 19 axes generated by CodonW using 20 amino acids. The total inertia of the amino acid usage is 0.004531. The explanation of the variation of Axis1 is 46.54% and Axis2 is 12.21%, others lower than 10%.

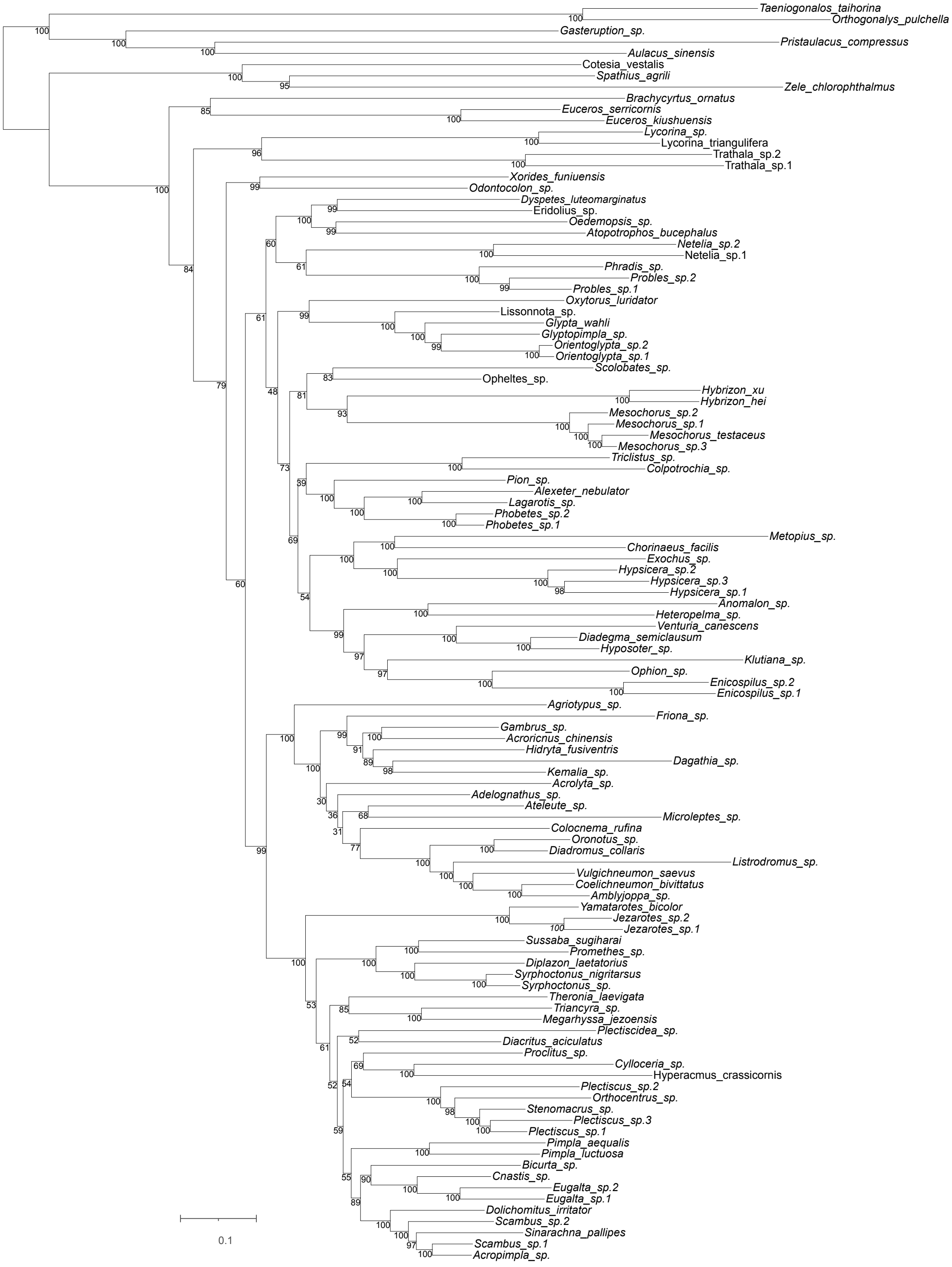

Figure S6 Phylogenetic relationships of Ichneumonid wasps inferred from amino acid sequences of 13 protein-coding genes in mitochondrial genomes (AA matrix) by ML method. Numbers close to the nodes are the ultrafast bootstrap values.

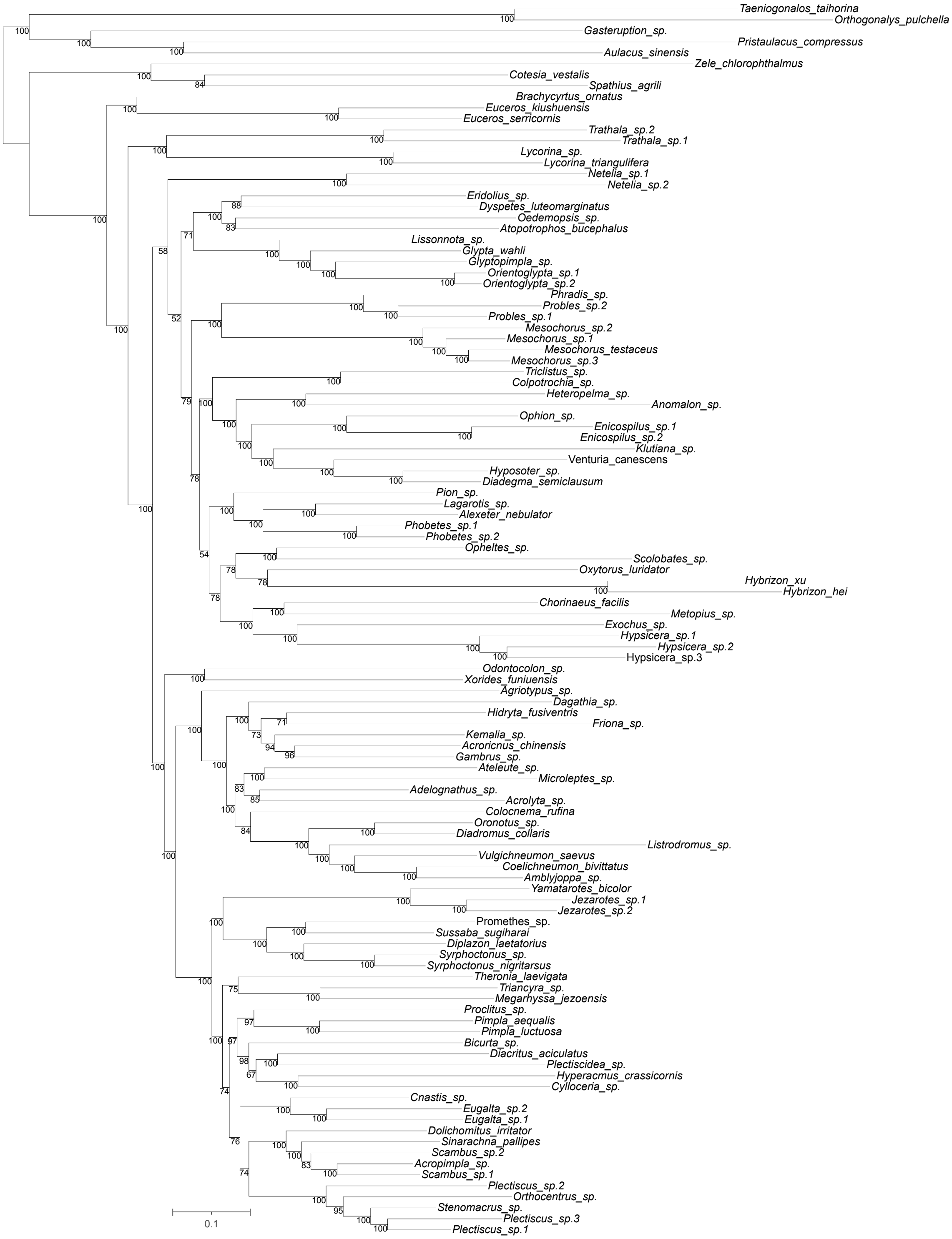
Taeniogonalos_taihorina
Orthogonalys_pulchella
Gasteruption_sp.
Pristaulacus_compressus
Aulacus_sinensis
Zele_chlorophthalmus
Cotesia_vestalis
Spathius_agrili
Brachycyrtus_ornatus
Euceros_kiushuensis
Euceros_serricornis
Trathala_sp.2
Trathala_sp.1
Lycorina_sp.
Lycorina_triangulifera
Netelia_sp.1
Netelia_sp.2
Eridolius_sp.
Dyspetes_luteomarginatus
Oedemopsis_sp.
Atopotrophos_bucephalus
Lissonnota_sp.
Glypta_wahli
Glyptopimpla_sp.
Orientoglypta_sp.1
Orientoglypta_sp.2
Phradis_sp.
Probles_sp.2
Probles_sp.1
Mesochorus_sp.2
Mesochorus_sp.1
Mesochorus_testaceus
Mesochorus_sp.3
Triclistus_sp.
Colpotrochia_sp.
Heteropelma_sp.
Anomalon_sp.
Ophion_sp.
Enicospilus_sp.1
Enicospilus_sp.2
Klutiana_sp.
Venturia_canescens
Hyposoter_sp.
Diadegma_semiclausum
Pion_sp.
Lagarotis_sp.
Alexeter_nebulator
Phobetes_sp.1
Phobetes_sp.2
Opheltes_sp.
Scolobates_sp.
Oxytorus_luridator
Hybrizon_xu
Hybrizon_hei
Chorinaeus_facilis
Metopius_sp.
Exochus_sp.
Hypsicera_sp.1
Hypsicera_sp.2
Hypsicera_sp.3
Odontocolon_sp.
Xorides_funiuensis
Agriotypus_sp.
Dagathia_sp.
Hidryta_fusiventris
Friona_sp.
Kemalia_sp.
Acroricnus_chinensis
Gambrus_sp.
Ateleute_sp.
Microleptes_sp.
Adelognathus_sp.
Acrolyta_sp.
Colocnema_rufina
Oronotus_sp.
Diadromus_collaris
Listrodromus_sp.
Vulgichneumon_saevus
Coelichneumon_bivittatus
Amblyjoppa_sp.
Yamatarotes_bicolor
Jezarotes_sp.1
Jezarotes_sp.2
Promethes_sp.
Sussaba_sugiharai
Diplazon_laetatorius
Syrphoctonus_sp.
Syrphoctonus_nigritarsus
Theronia_laevigata
Triancyra_sp.
Megarhyssa_jezoensis
Proclitus_sp.
Pimpla_aequalis
Pimpla_luctuosa
Bicurta_sp.
Diacritus_aciculatus
Plectiscidea_sp.
Hyperacmus_crassicornis
Cylloceria_sp.
Cnastis_sp.
Eugalta_sp.2
Eugalta_sp.1
Dolichomitus_irritator
Sinarachna_pallipes
Scambus_sp.2
Acropimpla_sp.
Scambus_sp.1
Plectiscus_sp.2
Orthocentrus_sp.
Stenomacrus_sp.
Plectiscus_sp.3
Plectiscus_sp.1
0.1

Figure S7 Phylogenetic relationships of Ichneumonid wasps inferred from nucleotide sequences of 13 protein-coding genes in mitochondrial genomes (NU matrix) by BI inference. Numbers close to the nodes are Bayesian posterior probabilities.

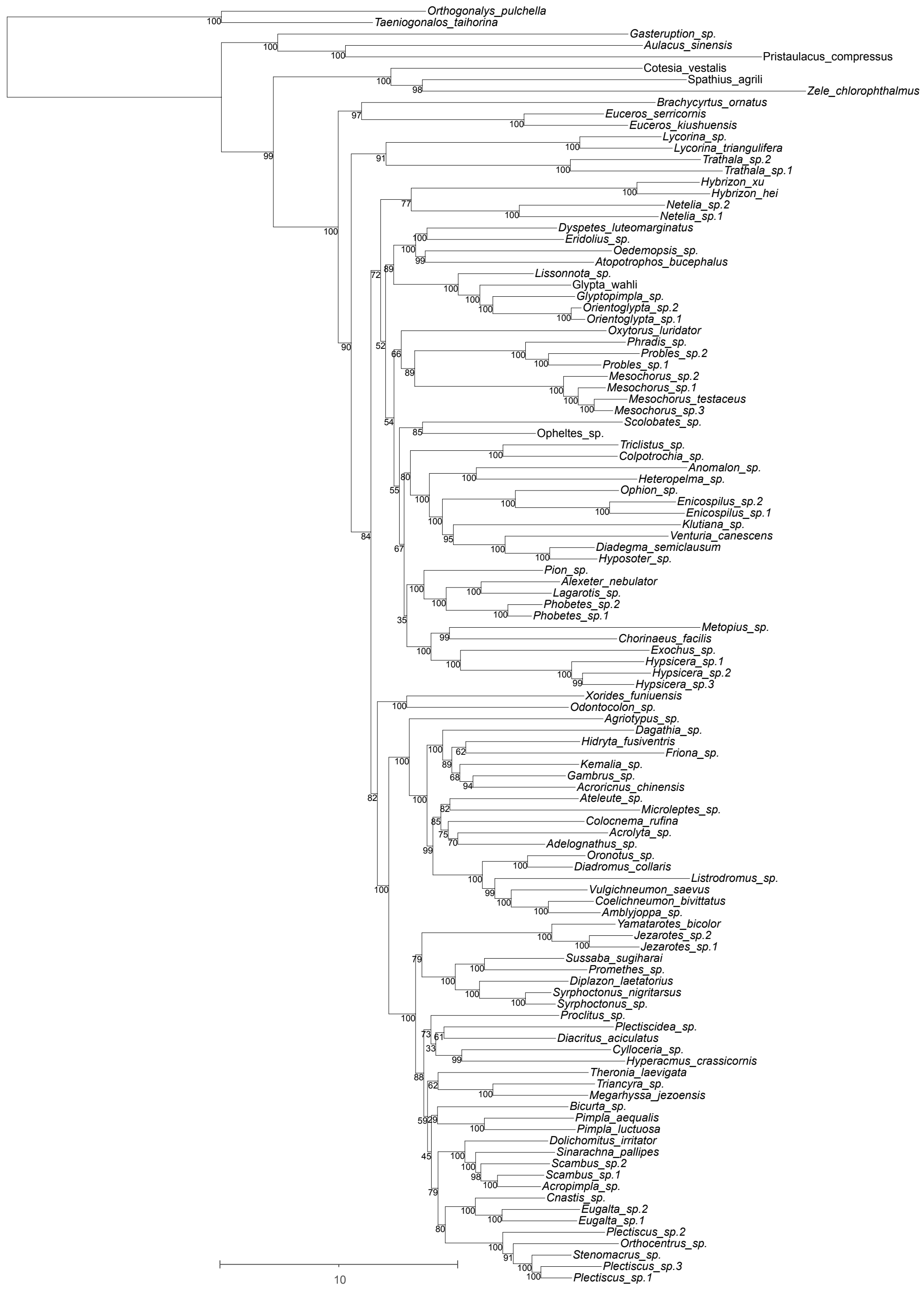

Figure S8 Phylogenetic relationships of Ichneumonid wasps inferred from nucleotide sequences of 13 protein-coding genes in mitochondrial genomes (NU matrix) by ML method.

Numbers close to the nodes are the ultrafast bootstrap values.

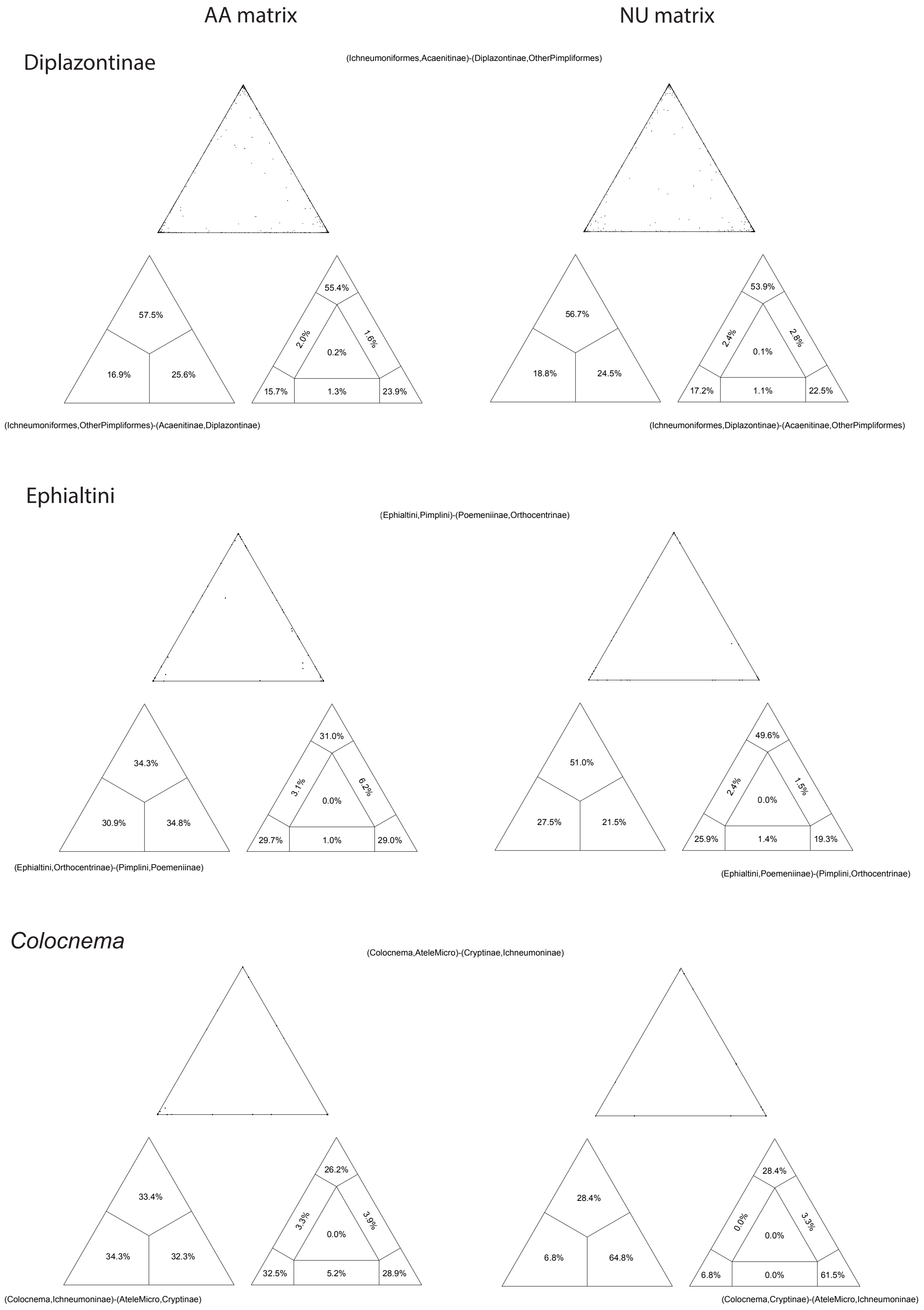

AA matrix
NU matrix
Diplazontinae
(Ichneumoniformes,Acaenitinae)-(Diplazontinae,OtherPimpliformes)
57.5%
16.9%
25.6%
55.4%
2.0%
1.6%
0.2%
15.7%
1.3%
23.9%
56.7%
18.8%
24.5%
53.9%
2.4%
2.8%
0.1%
17.2%
1.1%
22.5%
(Ichneumoniformes,OtherPimpliformes)-(Acaenitinae,Diplazontinae)
(Ichneumoniformes,Diplazontinae)-(Acaenitinae,OtherPimpliformes)
Ephialtini
(Ephialtini,Pimplini)-(Poemeniinae,Orthocentrinae)
34.3%
30.9%
34.8%
31.0%
3.1%
6.2%
0.0%
29.7%
1.0%
29.0%
51.0%
27.5%
21.5%
49.6%
2.4%
1.5%
0.0%
25.9%
1.4%
19.3%
(Ephialtini,Orthocentrinae)-(Pimplini,Poemeniinae)
(Ephialtini,Poemeniinae)-(Pimplini,Orthocentrinae)
Colocnema
(Colocnema,AteleMicro)-(Cryptinae,Ichneumoninae)
33.4%
34.3%
32.3%
26.2%
3.3%
3.9%
0.0%
32.5%
5.2%
28.9%
28.4%
6.8%
64.8%
28.4%
0.0%
3.3%
0.0%
6.8%
0.0%
61.5%
(Colocnema,Ichneumoninae)-(AteleMicro,Cryptinae)
(Colocnema,Cryptinae)-(AteleMicro,Ichneumoninae)

continued

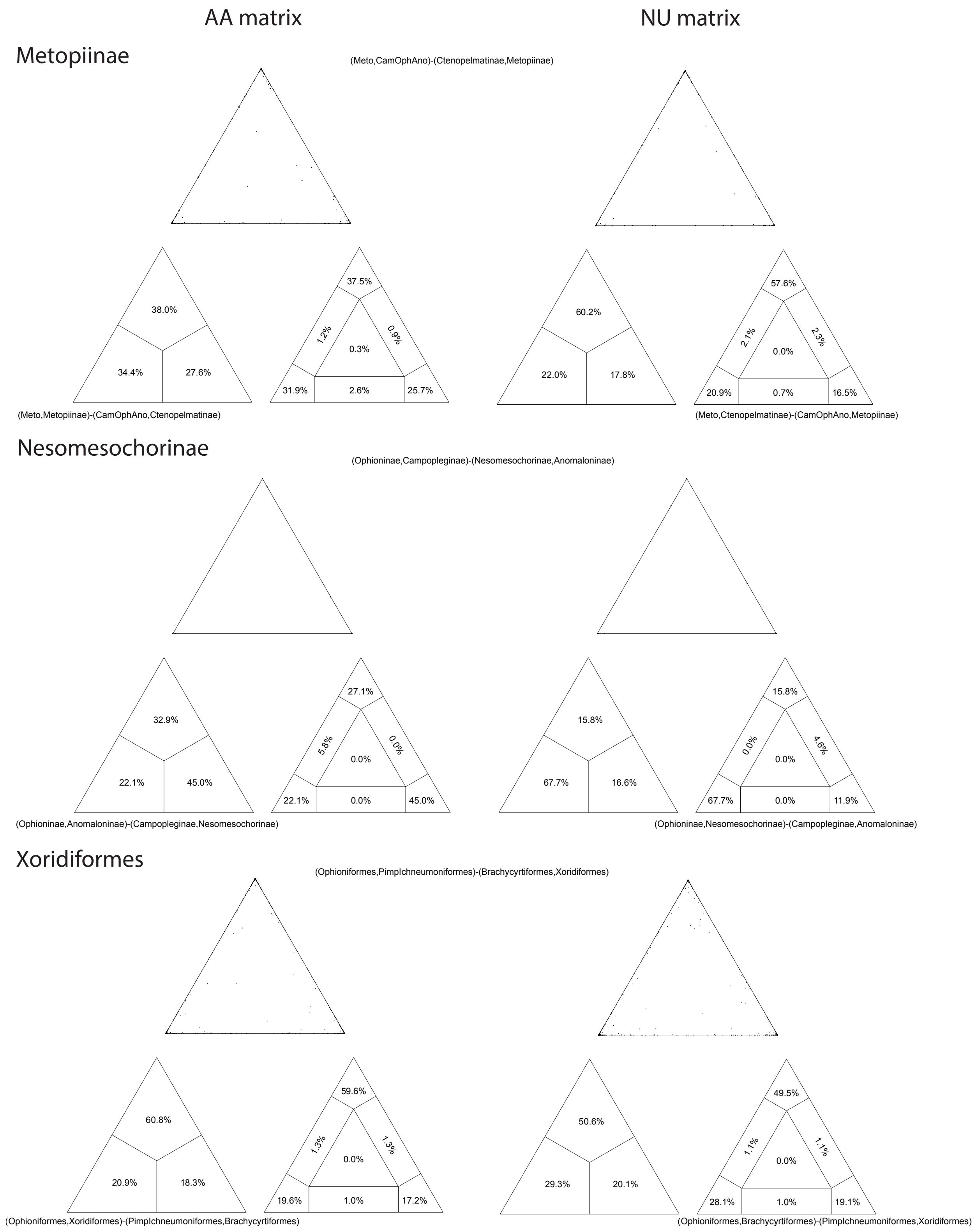

AA matrix
NU matrix
Metopiinae
(Meto,CamOphAno)-(Ctenopelmatinae,Metopiinae)
38.0%
34.4%
27.6%
37.5%
1.2%
0.9%
0.3%
31.9%
2.6%
25.7%
60.2%
22.0%
17.8%
57.6%
2.1%
2.3%
0.0%
20.9%
0.7%
16.5%
(Meto,Metopiinae)-(CamOphAno,Ctenopelmatinae)
(Meto,Ctenopelmatinae)-(CamOphAno,Metopiinae)
Nesomesochorinae
(Ophioninae,Campopleginae)-(Nesomesochorinae,Anomaloninae)
32.9%
22.1%
45.0%
27.1%
5.8%
0.0%
0.0%
22.1%
0.0%
45.0%
15.8%
67.7%
16.6%
15.8%
0.0%
4.6%
0.0%
67.7%
0.0%
11.9%
(Ophioninae,Anomaloninae)-(Campopleginae,Nesomesochorinae)
(Ophioninae,Nesomesochorinae)-(Campopleginae,Anomaloninae)
Xoridiformes
(Ophioniformes,PimpIchneumoniformes)-(Brachycyrtiformes,Xoridiformes)
60.8%
20.9%
18.3%
59.6%
1.3%
1.3%
0.0%
19.6%
1.0%
17.2%
50.6%
29.3%
20.1%
49.5%
1.1%
1.1%
0.0%
28.1%
1.0%
19.1%
(Ophioniformes,Xoridiformes)-(PimpIchneumoniformes,Brachycyrtiformes)
(Ophioniformes,Brachycyrtiformes)-(PimpIchneumoniformes,Xoridiformes)

continued

AA matrix NU matrix

Lycorininae and Cremastinae

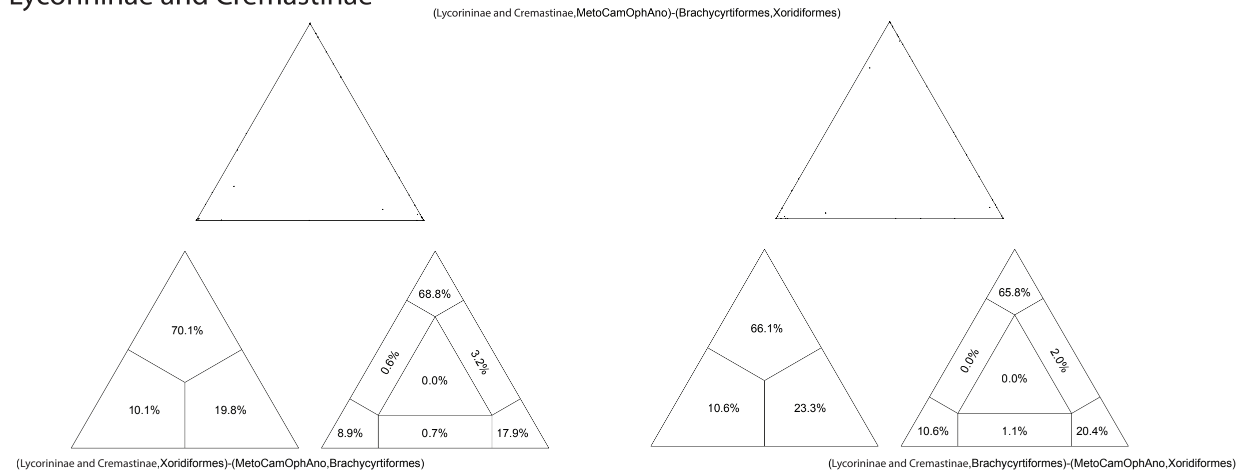

Lycorininae and Cremastinae

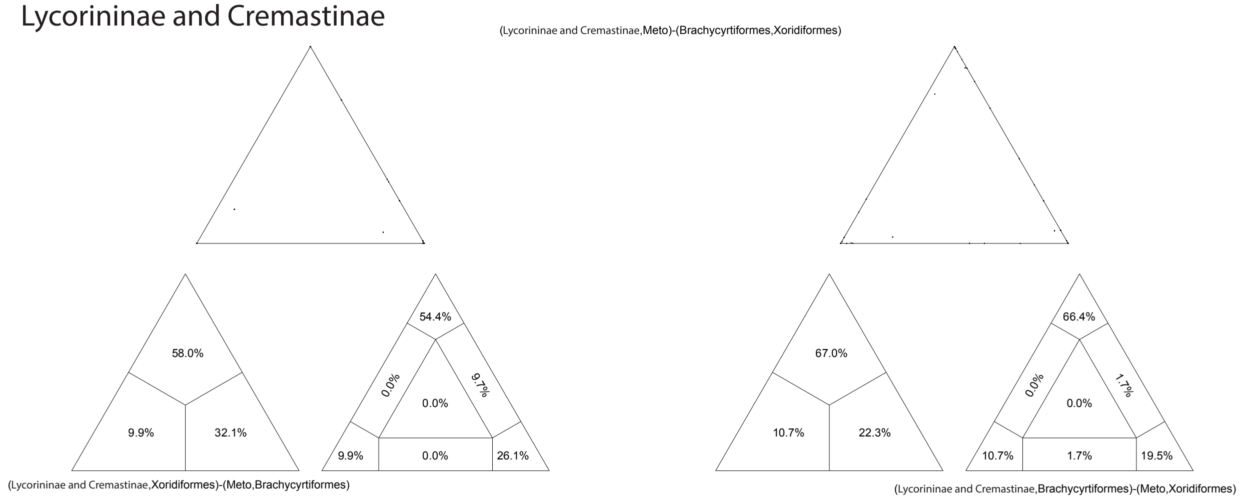

Lycorininae and Cremastinae

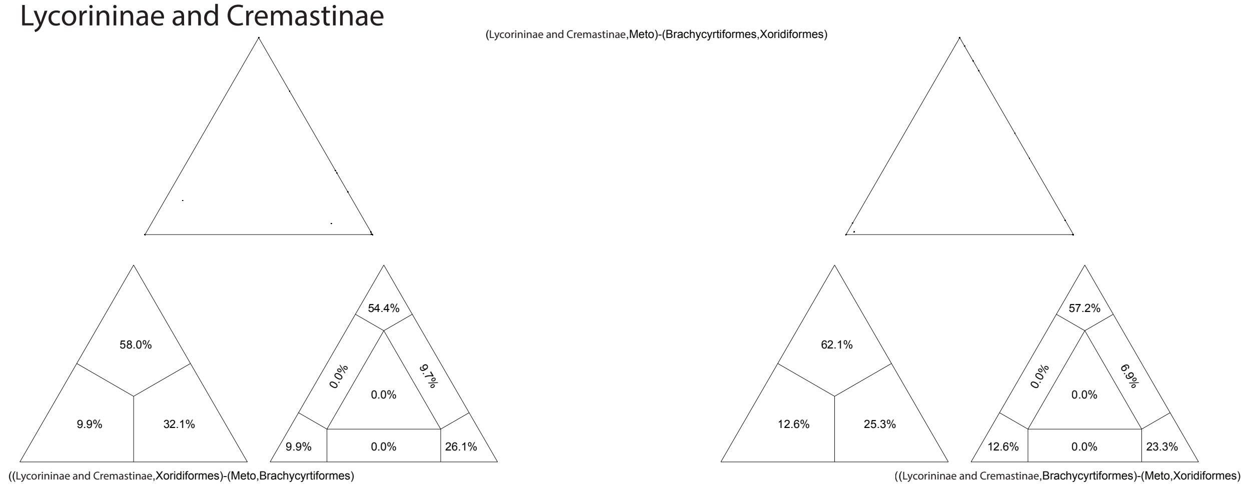

Figure S9 Results from likelihood mapping. The analysis was based on both AA and NU matrixes.

For per analysis of conflict nodes, the conflict nodes in tests are at the top right corners of rectangles, eg. the first test is for testing the placement of Diplazontinae. Distribution of quartets depicted by dots on the likelihood mapping plot (On the top). The three areas show support for one of the different groupings (On the left). The quartets falling into the three corners are informative (On the right). The support values were drawed at corresponding regions, which indicate three hypotheses for the relationship of four groups. The details for different groups was in Supplementary files. Groups abbreviation: OtherPimpliformes: Pimpliformes exclude Acaenitinae and Diplazontinae; AteleMicro: *Ateleute* sp. and *Microleptes* sp.; CamOphAno: 4 subfamilies include Anomaloninae, Nesomesochorinae, Campopleginae and Ophioninae; Meto: *Triclistus* sp. and *Colpotrochia* sp.; Ophioniformes: this high-level grouping here not include Lycorininae and Cremastinae; MetoCamOphAno: 4 subfamilies include Anomaloninae, Nesomesochorinae, Campopleginae, Ophioninae and Meto (*Triclistus* sp. and *Colpotrochia* sp.).

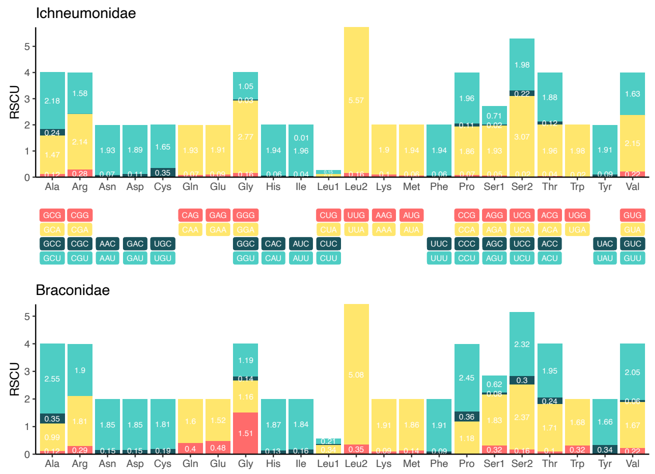

Fig. S10 The mean for relative synonymous codon usage (RSCU) of 13 protein-coding genes in mitochondrial genomes of Ichneumonidae and Braconidae.

The terminal codon is excluded. Different colors represent the 3rd sites of G (red), A (yellow), C (atrovirens) and U (blue).

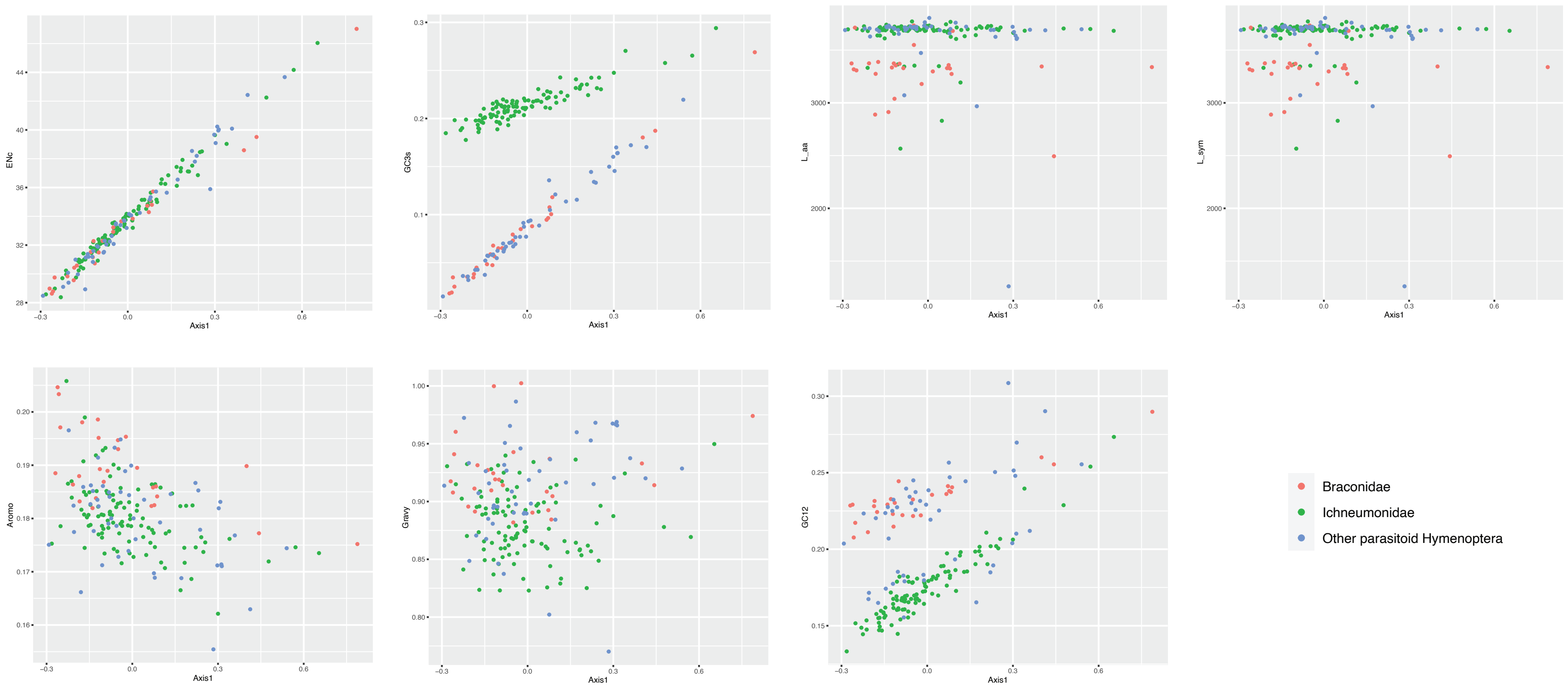

Figure S11 The Correlation test for the factors that influence RSCU bias against the first major axis (Axis1/48.93% explanation of the variation) generated by Correspondence Analysis (COA) in Ichneumonidae, Braconidae and other parasitoid Hymenoptera.

The factors include ENc (effective number of codons), GC3s (the G + C content at the 3rd codon positions, GC12 (the mean for the of G + C content at the 1st and 2nd codon positions); Aromo (general average aromaticity); GRAVY (general average hydropathicity); L_sym (length of synonymous codons); L_aa (length of amino acids).

Supplementary Reference

1. Tang, P.; Zhu, J.C.; Zheng, B.Y.; Wei, S.J.; Sharkey, M.; Chen, X.X.; Vogler, A.P. Mitochondrial phylogenomics of the Hymenoptera. *Mol. Phylogenet. Evol.* **2019**, *131*, 8–18, doi:10.1016/j.ympev.2018.10.040.
2. Wei, S.; Shi, M.; Sharkey, M.J.; Achterberg, C. van; Chen, X. Comparative mitogenomics of Braconidae (Insecta : Hymenoptera) and the phylogenetic utility of mitochondrial genomes with special reference to Holometabolous insects. *BMC Genomics* **2010**, *11*, 1471–2164, doi:10.1186/1471-2164-11-371.
3. Wei, S.; Shi, M.; He, J.; Sharkey, M.; Chen, X. The complete mitochondrial genome of *Diadegma semiclausum* (Hymenoptera : Ichneumonidae) indicates extensive independent evolutionary events. *Genome* **2009**, *52*, 308–319, doi:10.1139/G09-008.
4. Li, Q.; Wei, S.; Shi, M.; Chen, X.; Li, Q.; Wei, S.; Shi, M.; Chen, X. The mitochondrial genome of *Diadromus collaris* (Hymenoptera: Ichneumonidae). *Mitochondrial DNA* **2015**, *26*, 303–304, doi:10.3109/19401736.2013.825786.
5. Dowton, M.; Cameron, S.L.; Austin, A.D.; Whiting, M.F. Phylogenetic approaches for the analysis of mitochondrial genome sequence data in the Hymenoptera - A lineage with both rapidly and slowly evolving mitochondrial genomes. *Mol. Phylogenet. Evol.* **2009**, *52*, 512–519, doi:10.1016/j.ympev.2009.04.001.
6. Mao, M.; Gibson, T.; Dowton, M. Evolutionary dynamics of the mitochondrial genome in the Evaniomorpha (Hymenoptera)-A group with an intermediate rate of gene rearrangement. *Genome Biol. Evol.* **2014**, *6*, 1862–1874, doi:10.1093/gbe/evu145.
7. Wei, S.J.; Wu, Q.L.; Van Achterberg, K.; Chen, X.X. Rearrangement of the nad1 gene in *Pristaulacus compressus* (Spinola) (Hymenoptera: Evanioidea: Aulacidae) mitochondrial genome. *Mitochondrial DNA* **2013**, *26*, 629–630, doi:10.3109/19401736.2013.834436.
8. Wu, Q.L.; Li, Q.; Gu, Y.; Shi, B.C.; van Achterberg, C.; Wei, S.J.; Chen, X.X. The complete mitochondrial genome of *Taeniogonalos taihorina* (Bischoff) (Hymenoptera: Trigonalyidae) reveals a novel gene rearrangement pattern in the Hymenoptera. *Gene* **2014**, *543*, 76–84, doi:10.1016/j.gene.2014.04.003.
9. Mao, M.; Dowton, M. Complete mitochondrial genomes of *Ceratobaeus* sp. and *Idris* sp. (Hymenoptera: Scelionidae): shared gene rearrangements as potential phylogenetic

markers at the tribal level. *Mol. Biol. Rep.* **2014**, *41*, 6419–6427, doi:10.1007/s11033-014-3522-x.

10. Mao, M.; Austin, A.D.; Johnson, N.F.; Dowton, M. Coexistence of minicircular and a highly rearranged mtDNA molecule suggests that recombination shapes mitochondrial genome organization. *Mol. Biol. Evol.* **2014**, *31*, 636–644, doi:10.1093/molbev/mst255.
11. Wei, S.J.; Tang, P.; Zheng, L.H.; Shi, M.; Chen, X.X. The complete mitochondrial genome of *Evania appendigaster* (Hymenoptera: Evaniidae) has low A+T content and a long intergenic spacer between atp8 and atp6. *Mol. Biol. Rep.* **2010**, *37*, 1931–1942, doi:10.1007/s11033-009-9640-1.
12. Mao, M.; Gibson, T.; Dowton, M. Higher-level phylogeny of the Hymenoptera inferred from mitochondrial genomes. *Mol. Phylogenet. Evol.* **2015**, *84*, 34–43, doi:10.1016/j.ympev.2014.12.009.
13. Oliveira, D.S.; Gomes, T.M.F.F.; Loreto, E.L.S. The rearranged mitochondrial genome of *Leptopilina boulardi* (Hymenoptera: Figitidae), a parasitoid wasp of *Drosophila*. *Genet. Mol. Biol.* **2016**, *39*, 611–615, doi:10.1590/1678-4685-GMB-2016-0062.
14. Xiao, J.H.; Jia, J.G.; Murphy, R.W.; Huang, D.W. Rapid evolution of the mitochondrial genome in chalcidoid wasps (hymenoptera: Chalcidoidea) driven by parasitic lifestyles. *PLoS One* **2011**, *6*, doi:10.1371/journal.pone.0026645.
15. Dowton, M.; Cameron, S.L.; Dowavic, J.I.; Austin, A.D.; Whiting, M.F. Characterization of 67 mitochondrial tRNA gene rearrangements in the Hymenoptera suggests that mitochondrial tRNA gene position is selectively neutral. *Mol. Biol. Evol.* **2009**, *26*, 1607–1617, doi:10.1093/molbev/msp072.
16. Mao, M.; Valerio, A.; Austin, A.D.; Dowton, M.; Johnson, N.F. The first mitochondrial genome for the wasp superfamily Platygastroidea: The egg parasitoid *Trissolcus basalis*. *Genome* **2012**, *55*, 194–204, doi:10.1139/g2012-005.
17. Castro, L.R.; Ruberu, K.; Dowton, M. Mitochondrial genomes of *Vanhornia eucnemidarum* (Apocrita: Vanhorniidae) and Primeuchroeus spp. (Aculeata: Chrysididae): Evidence of rearranged mitochondrial genomes within the Apocrita (Insecta: Hymenoptera). *Genome* **2006**, *49*, 752–766, doi:10.1139/G06-030.
18. Li, Q.; Wei, S.J.; Tang, P.; Wu, Q.; Shi, M.; Sharkey, M.J.; Chen, X.X. Multiple lines of evidence from mitochondrial genomes resolve phylogenetic relationships of parasitic

wasps in Braconidae. *Genome Biol. Evol.* **2016**, *8*, 2651–2662, doi:10.1093/gbe/evw184.
